# Safe Healthcare Facilities: A Systematic Review on the Costs of Establishing and Maintaining Environmental Health in Facilities in Low- and Middle-Income Countries

**DOI:** 10.3390/ijerph18020817

**Published:** 2021-01-19

**Authors:** Darcy M. Anderson, Ryan Cronk, Donald Fejfar, Emily Pak, Michelle Cawley, Jamie Bartram

**Affiliations:** 1 The Water Institute, Gillings School of Global Public Health, University of North Carolina at Chapel Hill, Chapel Hill, NC 27599, USA; donlukef@live.unc.edu (D.F.); epak@live.unc.edu (E.P.); jbartram@email.unc.edu (J.B.); 2 ICF International, Durham, NC 27713, USA; ryan.cronk@icf.com; 3 Health Sciences Library, University of North Carolina at Chapel Hill, Chapel Hill, NC 27599, USA; mcawley@email.unc.edu; 4 School of Civil Engineering, University of Leeds, Leeds LS2 9JT, UK

**Keywords:** healthcare facilities, environmental health, water sanitation and hygiene, WaSH, waste management, cleaning, infection prevention and control, costing, finance, economic

## Abstract

A hygienic environment is essential to provide quality patient care and prevent healthcare-acquired infections. Understanding costs is important to budget for service delivery, but costs evidence for environmental health services (EHS) in healthcare facilities (HCFs) is lacking. We present the first systematic review to evaluate the costs of establishing, operating, and maintaining EHS in HCFs in low- and middle-income countries (LMICs). We systematically searched for studies costing water, sanitation, hygiene, cleaning, waste management, personal protective equipment, vector control, laundry, and lighting in LMICs. Our search yielded 36 studies that reported costs for 51 EHS. There were 3 studies that reported costs for water, 3 for sanitation, 4 for hygiene, 13 for waste management, 16 for cleaning, 2 for personal protective equipment, 10 for laundry, and none for lighting or vector control. Quality of evidence was low. Reported costs were rarely representative of the total costs of EHS provision. Unit costs were infrequently reported. This review identifies opportunities to improve costing research through efforts to categorize and disaggregate EHS costs, greater dissemination of existing unpublished data, improvements to indicators to monitor EHS demand and quality necessary to contextualize costs, and development of frameworks to define EHS needs and essential inputs to guide future costing.

## 1. Introduction

A safe, hygienic healthcare environment is important to provide quality patient care and prevent healthcare-acquired infections (HAIs). HAIs are the leading cause of preventable morbidity and mortality among hospitalized patients in high-income countries [1]. Although few data exist from low- and middle-income countries (LMICs), the burden of HAIs is likely higher where there are inadequate environmental health services (EHS) [2]. Systematic reviews of HAIs in Africa [3] and LMICs worldwide [2] estimate that approximately 15% of hospitalized patients acquire an HAI and that one in every 17 patients with an HAI dies from related causes [4]. HAIs are also associated with increased length of stay and result in billions of dollars spent annually on preventable medical expenses [5].

Environmental contamination is a substantial contributor to the burden of HAIs. An estimated 40–60% of HAIs are transmitted on the hands of healthcare workers, and an additional 20% are attributable to environmental contamination [6]. The importance of surface decontamination and environmental cleanliness for prevention of HAIs is well documented [7]. Adequate EHS are critical for safe care delivery and preventing HAIs [8,9] and may also improve patient satisfaction and reduce barriers to care seeking [10]. Efforts to better integrate environmental health in the health sector are an important intervention to reduce global maternal and newborn mortality [11,12,13].

The importance of EHS in healthcare facilities (HCFs) is recognized in international development policy though the Sustainable Development Goals (SDGs) with Goal 3 on health and Goal 6 on water, sanitation, and hygiene (WaSH). Goal 6 calls for universal access to WaSH, where “universal” is defined as including HCFs, as interpreted by the World Health Organization (WHO) and the United Nations Children’s Fund Joint Monitoring Programme (JMP) for Water Supply, Sanitation, and Hygiene, which has the responsibility for monitoring corresponding indicators under Goal 6 [14]. Appropriate EHS are necessary for a hygienic environment and to minimize risk of HAIs [15]. In response, the JMP developed indicators for monitoring healthcare settings that comprise hygiene availability to healthcare providers at the point of care, medical waste disposal, and surface cleaning in maternity wards [16]. The WHO’s standards for essential environmental health in HCFs include food hygiene; laundry; energy and lighting; ventilation, heating and cooling; and control of arthropod, rodent, and other animal disease vectors [17].

HCFs have been identified as an area of urgent need by the United Nations Secretary General to meet the SDGs by 2030 [18]. An estimated 50% of HCFs in LMICs lack piped water on premises, 33% lack improved sanitation, 39% lack handwashing soap, 39% lack adequate infectious waste disposal, and 73% lack sterilization equipment [19]. Other EHS not monitored under the JMP are similarly insufficient. Approximately 26% of HCFs in sub-Saharan Africa lack access to a reliable electricity supply [20].

Barriers to achieving universal access to adequate EHS in HCFs include lack of political will and funding. Of the countries included in the WHO’s Global Analysis and Assessment of Sanitation and Drinking-Water, approximately half have developed and costed a national implementation policy for hygiene promotion (52%), sanitation and drinking water (58%), and infection prevention and control (57%) in HCFs. However, only 22% have a financing plan in place for WaSH in HCFs that is consistently implemented [21]. In a survey of 2035 HCFs in 14 countries in Asia, Africa, and Latin America, less than half of all facilities in each country reported sufficient budget allocated for infection prevention and control and WaSH supplies [22].

Understanding costs can help improve EHS delivery through better informed budgeting and decision-making, for example, through informing the selection of sustainable and cost-effective modalities of EHS provision [23]. However, costs of EHS provision in HCFs in LMICs are poorly understood and considered a priority for future work on improving basic services [15]. Paucity of costs data has led to calls for greater collection and application of evidence to inform investment in EHS in HCFs [15,24], yet a corresponding response has been lacking. Previous research has proposed models to support budgeting for EHS in HCFs in LMICs [25], which have been applied for costing in case studies in a narrow range of contexts [26], but evidence of costs across a wide variety of LMIC settings has not been comprehensively documented.

In this paper, we present the first systematic review on the costs of EHS in HCFs in LMICs. The purpose of this review is to evaluate the costs of establishing, operating, and maintaining adequate EHS provision in LMICs in nine focus areas identified in the WHO’s *Essential Environmental Health Standards in Healthcare* [17,18]: water, sanitation, hygiene, waste management, surface and medical device cleaning and sterilization (hereafter referred to as “cleaning”), personal protective equipment (PPE), laundry, lighting, and control of arthropod, rodent, and other animal disease vectors (“vector control”). We assess these costs in low-income, lower-middle income, and upper-middle income countries following the World Bank’s income group classifications [27].

## 2. Methods

### 2.1. Search Strategy and Eligibility Criteria

We conducted this systematic review following the Preferred Reporting Items for Systematic Reviews and Meta-Analyses (PRISMA) guidelines [28]. The PRISMA checklist is included as Supplementary Information File 1. Specific procedures are described below.

We developed a protocol a priori, which is provided in Supplementary Information File 2. We initially searched PubMed, EBSCO (Global Health and Business Source Premier), Scopus, and Web of Science from inception to 5 November 2017. We updated our search on 24 September 2019. In the update, we included the ProQuest Theses and Dissertations Global database, also searched from inception. We included publications not captured by our search recommended by experts within the field and searched the reference lists of included publications for relevant papers. Databases were searched using Boolean operators for at least one term from four clusters: environmental health and infection prevention and control; HCFs; costing, accounting, and financial analysis; and countries classified as low, lower-middle, or upper-middle income in the World Bank’s historical income group classification [27]. Search term development and database selection were informed by recent reviews of WaSH in HCFs and HAIs [15], care-seeking behavior and healthcare satisfaction [10], and the costs of WaSH in schools [29]. For details on the search strategy, see Supplementary Information File 3.

We considered the following as EHS: water, sanitation (including storm and other greywater management), waste management, cleaning, PPE, laundry, lighting, and vector control. We defined HCFs as any permanent institution with the primary purpose of delivering medical services. Studies of healthcare delivered in institutions whose primary purpose was nonmedical, such as schools or residential group homes, and medical research facilities that did not see patients were excluded. We also excluded nonpermanent facilities, such as temporary facilities established for vaccination campaigns and mobile clinics. HCF search terms included both general and specialized facilities, such as dental and health facilities providing maternal and newborn healthcare.

We defined eligible costs based on the principle of lifecycle costing. Lifecycle costing considers the entire “lifecycle” of a technology—from installation through operations and maintenance and finally decommissioning and disposal—and the expenses needed within each phase of the lifecycle [30]. Costs across all phases of the lifecycle were eligible for inclusion.

We conducted all searches in English. EBSCO, Scopus, Web of Science, and ProQuest Theses and Dissertations Global were searched for titles, abstracts, and keywords. In PubMed, terms were searched in all fields and automatic term mapping was applied. Search results were exported to Covidence reference management software (Veritas Health Innovation, Melbourne, Australia) for deduplication and screening. Two reviewers independently screened all publications and resolved disputes by discussion.

We included English, French, and Spanish language studies. Studies were included if they evaluated the costs of providing an eligible EHS in an HCF in a low-, lower-middle, or upper-middle-income country. We used the World Bank historical income classifications for countries based on the year in which studies were published. We included studies conducted at sub-facility-level, so long as environmental costs were evaluated for all care provided within the unit. We excluded studies that evaluated procedure-specific costs for only a subset of care provided. We also excluded studies that modeled hypothetical spending but did not conduct field data collection and studies where the only environmental cost was a utility bill. Where EHS data were ambiguous, collected but not reported, or aggregated with other noneligible expenses, we contacted authors to request disaggregated data. We excluded studies for which we received no response.

### 2.2. Search Update Strategy

We used machine learning to assist with title and abstract screening during the search update. Machine learning is particularly valuable for systematic review search updates, as it can use the results of manual screening in the original search as training data for machine learning algorithms to automate portions of the screening process for studies yielded in search updates, substantially reducing screening time and effort [31].

We used semi-supervised learning and supervised machine learning in two phases to prioritize studies from the search update for manual screening using the DoCTER software (Document Classification and Topic Extraction Resource) (ICF, Virginia, USA). All studies from the search update that were screened manually were reviewed according to the guidelines in our protocol (see Supplementary Information File 2).

DoCTER prioritizes search results using the text of titles and abstracts and has functions for clustering, supervised clustering, and supervised machine learning. We used supervised clustering with an ensemble approach for the initial round of prioritization. Supervised clustering is a form of semi-supervised learning that groups an unclassified corpus of studies with a set of known relevant (i.e., “seed”) studies. Clusters containing a high proportion of seed studies are expected to contain a high proportion of relevant studies and are prioritized for manual screening. Varghese, et al. [32] provides full details on supervised clustering and demonstrates that the method rivals accuracy rates of supervised machine learning algorithms while requiring less training data.

The ensemble approach uses two algorithms: k-means and nonnegative matrix factorization (NMF) and three cluster sizes: 10, 20, and 30. Using each algorithm with the three different cluster numbers yields six different clustering models (e.g., KM-10 model is the k-means algorithm with 10 clusters and KM-20 is the k-means algorithm with 20 clusters). The six models were applied to title and abstract text of the literature search update with a set of seed studies. Seed studies are a form of training data but require fewer positive studies than typically necessary for machine learning algorithms. Our seed set included the 154 relevant studies that were captured from title and abstract screening in the original search. Shekelle et al. [33] showed the efficacy of using training data from an initial systematic review to prioritize results from search updates and captured 96% of relevant studies while reducing the volume of manual screening by 78% on average. Cawley et al. [34] also demonstrated that externally-derived training data were effective at identifying relevant studies in an unclassified corpus. 

The output of supervised clustering with a six model ensemble approach is an ensemble score ranging from 0 to 6 for each study that indicates the number of models where the study was found in a relevant cluster (i.e., a cluster with a high proportion of seed studies). We manually screened studies with an ensemble score of 3 or higher. The remaining studies were sent to a second phase of prioritization using supervised machine learning. Training data were derived from the studies screened previously, and studies with a 0.5 or greater probability of relevance were screened manually.

### 2.3. Data Extraction

Two reviewers conducted data extraction for each study using a pilot-tested extraction form in Microsoft Excel (Redmond, Washington). Supplementary Information File 4 contains the final extraction form. A third reviewer compared results; we resolved disputes by discussion. Extracted data included: facility type, location, and indicators of patient volume; EHS description and included costs; costing methodology; and main findings of cost calculations. We coded costs as capital hardware, capital software, capital maintenance, consumables, personnel, recurrent training, direct support, financing, or contracted services (Table 1), following Anderson et al. [25].

Our primary summary measures were total EHS cost and sub-costs by categories described in Table 1, where possible to disaggregate. We converted all costs into United States dollars (USD) [35] adjusted for inflation to 1 October 2018. We used the conversion rate for the median of the data coverage period. For studies reporting years only, we used 1 January. For studies reporting no dates of data coverage, we used the publication date. Due to heterogeneity in technologies and approaches used for EHS provision, costing methodologies, and specific cost components included, we did not conduct a meta-analysis. Instead, we provide a narrative summary of costs.

### 2.4. Quality Assessment

We developed a tool to assess study quality informed by reporting guidelines for healthcare economic studies [36,37,38]. Two independent reviewers rated each study quality for each EHS as high (+1 point), moderate (+0.5 points), or low (0 points) quality for 12 items related to context reporting (costing objective, facility description, EHS quantity indicators, EHS quality indicators), costing reporting (units reporting, line item reporting, analysis reporting), and costing methodology (framework use, data sources, coverage duration, and cost category coverage). Where reviewers disagreed, disputes were resolved by discussion. For papers reporting on multiple EHS, each EHS was scored separately, as methods and reporting sometimes differed across EHS within a single study. Points were averaged across all 12 items for an overall quality score.

We define EHS quantity as the demand for a given EHS based on patient volume and other factors (e.g., whether services are inpatient or outpatient). We define EHS quality as the safety and sufficiency of the service. For example, the kilograms of waste produced at a facility per day requiring treatment is an indicator of EHS quantity, while the proportion of waste properly segregated or pathogen reduction from treatment are indicators of quality. For full definitions of other measures used in the quality assessment, Supplementary Information File 5 contains the scoring tool and criteria.

## 3. Results

Our search retrieved 46,964 publications (37,297 in the original search, 9667 in the update), 32,205 of which were unique. We manually screened titles and abstracts for all studies from the original search, as well as 880 studies with an ensemble score of 3 or higher identified in the first phase of machine learning and 1541 studies with a probability of relevance greater than 0.5 in the second phase of machine learning from the search update. The remainder were screened using supervised machine learning through DoCTER. Following title and abstract screening, we reviewed 235 full texts. Four studies were included from the search update in the final analysis. All were found in the initial phase of prioritization (i.e., supervised clustering with ensemble score of 3 or higher). None of the studies manually screened from the supervised machine learning step were found to be relevant after title-abstract screening and full-text review.

**Figure 1:**
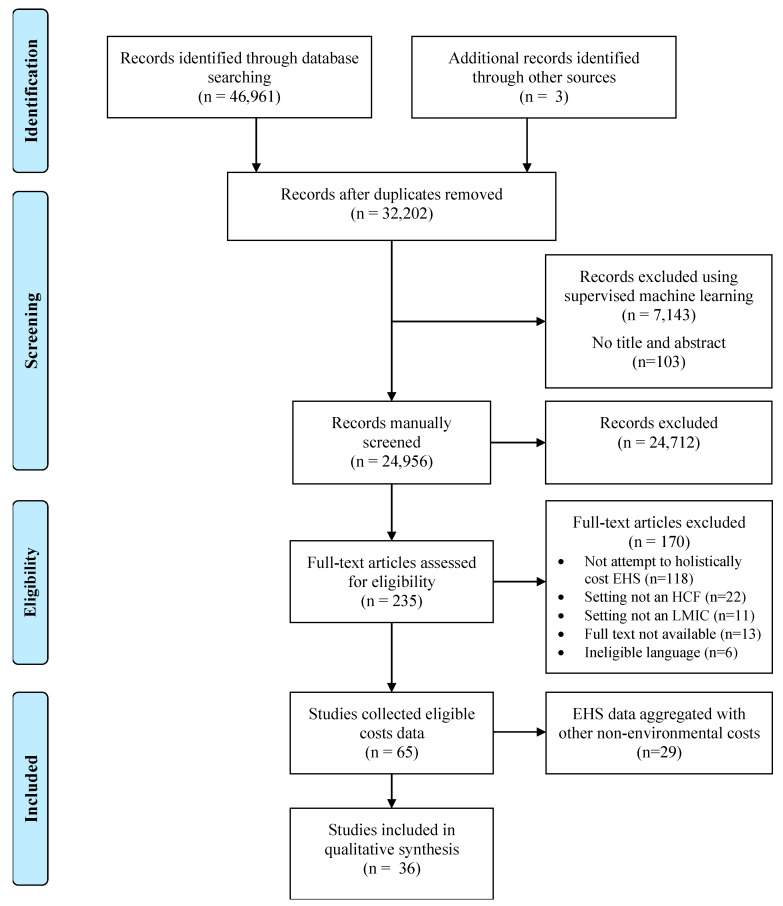
PRISMA flow chart of included studies.

We included 36 studies that reported disaggregated costs for 51 different EHS (Figure 1). Of the 51 EHS, 3 (6%) were costs for water, 3 (6%) for sanitation, 4 (8%) for hygiene, 13 (25%) for waste management, 16 (31%) for cleaning, 2 (4%) for PPE, and 10 (20%) for laundry. Our search retrieved data for 64 EHS across 29 studies where costs were collected but not disaggregated and/or reported: 17 for water, 4 for sanitation, 4 for waste management, 15 for cleaning, 20 for laundry, and 4 for lighting. We found no studies on vector control. For studies collecting but not reporting eligible costs data, EHS costs were most often combined with “overhead” expenses.

Studies represented 6 World Bank regions: East Asia and Pacific (n = 11 studies), South Asia (n = 11), Sub-Saharan Africa (n = 6), Latin America and the Caribbean (n = 3), the Middle East and North Africa (n = 3), and Europe and Central Asia (n = 2).

**Figure 2:**
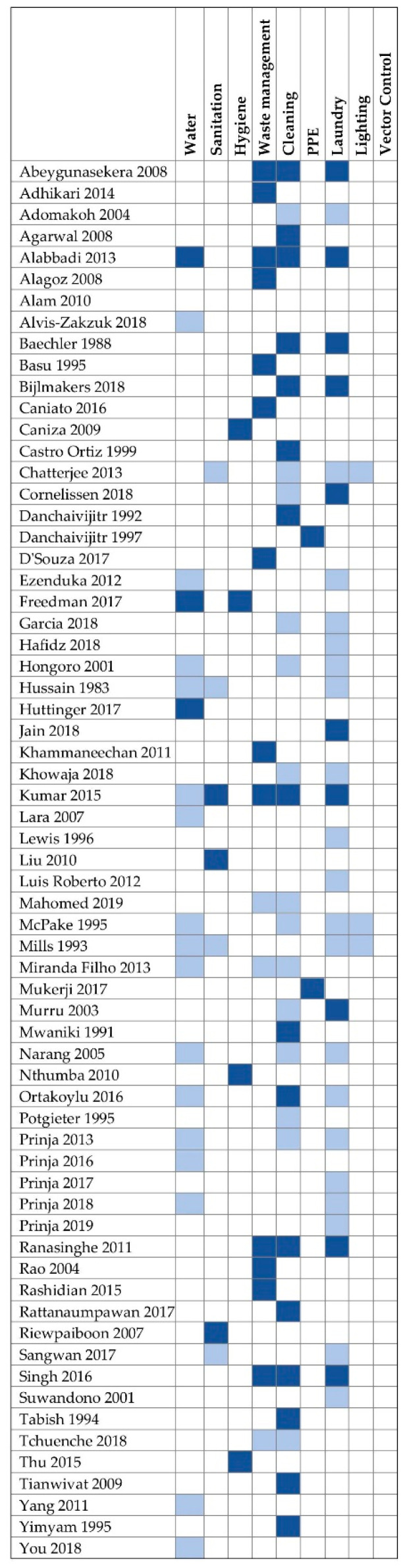
Evidence map of studies costing EHS. Dark blue cells indicate EHS for which costs data were collected and reported. Light blue cells indicate EHS for which costs data were collected but not reported or disaggregated. Non-highlighted cells indicate that costs data were neither collected nor reported. PPE = personal protective equipment.

Figure 2 depicts an evidence map of studies reporting and/or collecting eligible costs data and provides references for the studies included under each EHS. Supplementary Information File 4 contains extraction tables for all studies reporting costs data. Supplementary Information File 6 lists studies that collected but did not report disaggregated data.

**Figure 3:**
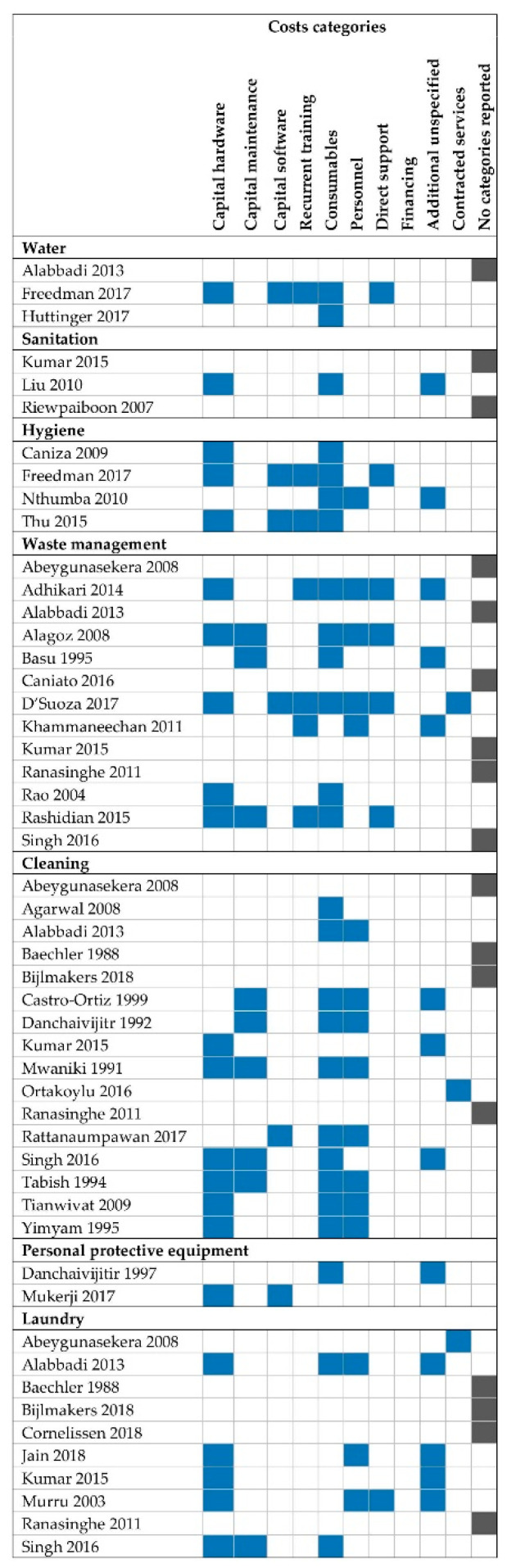
Cost category coverage of included studies. Blue cells indicate at least one line item from that costs category were included. Grey cells in the final column “Not reported” indicate that no line items were specified. “Additional unspecified” indicates that some line items were reported that could be categorized but costs also included other unspecified expenses.

Costing EHS was an explicit objective in 21 (41%) studies. The remainder costed EHS in the context of assessing costs of overall facility operation or as part of an intervention effectiveness evaluation. Specialized facilities—specifically surgical, urology, dialysis, trauma and intensive care units or centers—were frequent among included studies (n = 7 studies, 19%). Consumables (e.g., sharps containers, sterile gloves, fuel) were the most commonly included cost category (51% of EHS), followed by capital hardware (43%). Few studies included costs of capital software (12%), capital maintenance (18%), recurrent training (14%), or direct support (14%). Specific line items or cost categories were not reported for 17 EHS (Figure 3).

Most studies focused on costs of operating already established EHS (n = 29, 81%). Capital hardware costs most commonly included equipment installed after facility construction. Construction costs were only included for EHS provided in self-contained buildings that served multiple wards or units in large multi-building facilities: waste treatment facilities [39], central sterilization departments [40,41,42,43], and laundry facilities [40,43,44,45]. We found no studies that costed construction, rehabilitation, or upgrades to EHS integrated into facilities where direct patient care was provided.

Differences in the cost categories, including country contexts, facilities sizes, and other contextual factors, limited the ability to meaningfully compare summary measures of costs across included EHS. As such, subsequent sections present a narrative summary of findings for each EHS without summary tables. However, we direct the reader to Supplementary Information File 4, which includes the full extraction results for each study. The full extraction tables may be sorted by EHS, country, World Bank geographic region and income classification, and present cost findings alongside contextual narrative description on facility characteristics important for more nuanced comparisons.

### 3.1. Water

Our search yielded three studies reporting disaggregated costs for water. Freedman et al. [46] costed a combined intervention of portable drinking water and hygiene stations in 117 HCFs in rural Kenya. Program costs for water were USD 377 per facility and included hardware provision, training, procurement and distribution, and overhead costs, but excluded labor for facility restocking, maintenance, and consumable supplies for water treatment beyond an initial three-month supply. Alabbadi et al. [44] estimated USD 16,236 annually for water “overhead” for a 200-bed facility in Jordan but did not specify included expenses. Huttinger et al. [47] assessed costs of water kiosks installed at nine HCFs in Rwanda as small businesses, with water production costs of USD 0.02 per 20 liters of water and USD 16,500 per facility for initial purchase of ultramembrane filtration and chlorination devices for water treatment.

### 3.2. Sanitation

We found three studies reporting eligible sanitation costs. Kumar et al. [40] reported costs of sewage plant operation apportioned to one polytrauma and one specialized neurosurgery intensive care unit (ICU) in a level-IV trauma center in India at USD 0.15 and USD 0.09 per bed-day, respectively, but did not specify included expenses. Liu et al. [48] reported capital hardware and operating costs of biomembrane reactors at four different hospitals in China ranging from USD 0.23 to 0.62 per cubic meter of sewage. Riewpaboon et al. [49] costed sanitation for a district hospital in Thailand at USD 14,766 annually, with district hospitals in the region averaging 30–60 beds and no additional facility characteristics described. Expenses were not clearly described but may exclude maintenance, as “building maintenance” was costed as a separate category.

### 3.3. Hygiene

Four included studies costed hygiene. All were impact evaluations of hand hygiene interventions for medical providers that included a cost or cost-effectiveness component. We found no studies that costed hygiene services for patients. Caniza et al. [50] costed the installation of 41 alcohol hand rub dispensers and monthly use of hand rub in five wards of a 300-bed hospital in El Salvador at USD 2558 for dispenser installation and USD 731 monthly for hand rub costs. Nthumba et al. [51] compared the effectiveness of handwashing with soap versus hand rub for preventing HAIs in a surgical service in Kenya performing approximately 500 surgeries annually. Costs included procurement, preparation, and dispensing of alcohol-based hand rub, totaling USD 7.57 per week. Soap expenses were not specified but reported as USD 5.43 per week. Thu et al. [52] costed a hand hygiene intervention including the installation of sinks, paper towel dispensers, alcohol-based hand rubs, and hygiene promotion over a 10-month period in ICUs and critical care units at hospitals in Vietnam. Total costs were USD 14,091 and USD 7.29 per patient treated. Freedman et al. [46] costed portable handwashing facilities installed at 117 facilities in Kenya, including costs of hardware, training, procurement and distribution, and overhead, at an average of USD 377 per facility.

### 3.4. Healthcare Waste Management

Our review yielded 13 eligible studies for waste management. Five studies reported overall costs but not specific expenses. Kumar et al. [40] costed “biomedical waste disposal” in India at USD 0.28 and USD 0.17 per bed-day at a polytrauma and a neurosurgery ICU, respectively. Abeygunasekera et al. [53] costed “waste management and cleaning services” at USD 142 over a one-month study period for a 19-bed urology unit in Sri Lanka. Alabbadi et al. [44] reported waste treatment costs at a hospital in Jordan of USD 355,568 annually, or USD 3.25 per kilogram of waste treated. Ranasinghe et al. [54] costed waste management in five hemodialysis units in hospitals in Sri Lanka ranging from 3300 to 250 beds from USD 17 to 143 per month. Costs were not proportional to unit size, with the 3300-bed unit costing 17 USD per month and two 250-bed units costing USD 143 and 99 per month, each. Singh et al. [43,47] reported USD 84 per month for “biomedical waste management” in a 20-bed ward and six-bed ICU of a trauma center in India.

Four studies costed specific activities in the waste management process. Alagoz and Kocasoy [55] reported fixed costs of USD 3187 per day and variable costs of USD 0.11 per kilogram for waste transportation between a network of 24 hospitals and 18 clinics in Istanbul. Basu [56] costed operation and maintenance of an on-site incineration facility for a “large” teaching hospital in India at USD 4298 per month. Caniato et al. [57] costed waste disposal, excluding collection and treatment, of pharmaceutical wastes at 16 facilities in the West Bank at USD 48 per cubic meter of waste. Rashidian et al. [39] costed construction and equipment costs, as well as transportation, to nine waste treatment plants using different technologies in Iran. Costs were annualized over an assumed 10-year treatment technology lifespan and ranged from USD 97,904 to 612,864 annually, with hydroclave technologies being most cost-effective.

Three studies costed multiple waste management activities. D’Souza et al. [58] costed yearly segregation, internal transport, and outsourced final disposal in India for a 2032-bed hospital (USD 0.23 per bed-day), 268-bed hospital (USD 0.26 per bed-day), and 112-bed hospital (USD 0.37 per bed-day). Khammaneechan et al. [59] costed segregation collection, storage, and on-site incineration or transportation and off-site incineration for 127 facilities served by three different waste treatment plants in Thailand. Costs ranged from USD 4480 to 151,457 per facility per year. Rao et al. [60] costed collection, segregation, storage, transportation, and off-site disposal for five facilities in India. One-time costs for capital hardware purchases ranged from USD 1574 to USD 10,860 and recurrent costs ranged from USD 738 to USD 1066 per month.

Adhikiri and Supakankunit [61] costed a health care waste management intervention designed to reduce, recycle, and reuse typically disposed products and to improve segregation and safe management practices from source to final disposal at a hospital in Nepal. Costs totaled USD 38,774 per day for facility-wide costs and USD 135 per bed-day for capital hardware, consumables, labor, and training.

### 3.5. Cleaning and Sterilization

Our search yielded 16 eligible studies for cleaning. Four studies reported unspecified facility-wide cleaning costs. Alabbadi et al. [44] costed “cleaning supplies” and cleaner salaries for a 200-bed private facility in Jordan at USD 1,233,936 annually. Ranasinghe et al. [54] costed “cleaning services” for five hemodialysis units in Sri Lanka ranging from USD 30 to USD 182 per month. Bijlmakers et al. [62] costed “housekeeping and security” for surgery units in 278-bed and 325-bed public hospitals in Zambia at USD 2208 and USD 68,159 for one year, respectively. Ortakoylu et al. [63] report cleaning costs of USD 52,857 per year for a 400-bed hospital specializing in chest diseases and thoracic surgery in Turkey. Two studies combined costs of cleaning with other EHS. Abeygunasekera et al. [53] combined cleaning and waste disposal as a single aggregated service but did not specify a costing methodology or specific expenses. Baechler and Ortiz [64] combined costs of “cleaning and laundry supplies” at USD 207 annually for a rural health post in Cuba.

Five studies costed the operation of central sterilization departments. Castro-Ortiz [65] costed a central sterilization department serving a hospital of unknown size in Santiago, Cuba at USD 1030 over a three-month study period. Kumar et al. [40] costed building, equipment, and unspecified “operating” expenses at USD 6.25 per bed-day for both a 22-bed polytrauma and a 20-bed neurosurgery ICU in India. Singh et al. [43,44] apportioned USD 15,257 annually for operation of a central sterilization department to a 20-bed ward and 6-bed ICU in an Indian trauma center. Tabish et al. [41] costed consumables, labor, and maintenance of a 6000 square foot sterilization facility in India at USD 73,405 annually, with a USD 27,120 one-time investment in an autoclave. Tianviwat et al. [42,48] costed the operation of a cleaning and sterilization unit in a 30-bed Thai hospital at USD 1402 over a three month study period.

Three studies costed processing of reusable syringes [66,67,68]. Costs per syringe recycled ranged from USD 0.09 to 0.29. Higher estimates included a more comprehensive range of costs, including sterilization facility construction, which were excluded from lower cost estimates. Two studies costed other specific interventions. Agarwal et al. [69] reported costs of antiseptic and disinfectant use from before and after an intervention to promote proper disinfection procedures in a 120-bed hospital in India, totaling USD 8203 in the preintervention year and USD 5505 in the postintervention year. Rattanaumpawan and Thamlikitul [70] costed an individualized infection control intervention implemented in medical wards in a 2200-bed Thai hospital, including protective equipment and salaries for infection control nurses, at USD 5.50 per hospitalization day.

### 3.6. Personal Protective Equipment

Two included studies costed PPE. Danchaivijitir et al. [71] costed packing, cleaning, sterilization, and redistribution of reusable PPE at USD 3,852,340 for combined costs across 24 Thai government hospitals. Per item costs of recycling ranged from USD 0.02 per apron to USD 0.50 per pair of sterile gloves. Mukerji et al. [72] costed respirator use for six treatment arms in a trial comparing performance of fit tested versus nonfit tested N95 respirators or medical masks in Chinese hospitals. Costs ranged from USD 72 per 28-day study period for continuous use of fit-tested N95 masks to USD 11 for continuous use of medical masks.

### 3.7. Laundry

Our search yielded ten eligible studies for laundry. Seven studies reported costs for self-contained laundry facilities or contracted services. Alabbadi et al. [44] costed the construction (USD 162,360 for a one-time investment), annual operation (USD 129,888), and annual worker salaries (USD 116,899) for a laundry facility serving a 200-bed private hospital in Jordan. Abeygunasekera et al. [53] reported USD 111 for a one-month laundry contract for a 19-bed urology unit in Sri Lanka. Murru et al. [45] costed the laundry facility operation for a 468-bed teaching hospital in Uganda at USD 56,732 annually for labor, building, and equipment costs. Singh et al. [43,47] costed the construction and operation of a laundry facility for a 20-bed disaster facility and 6-bed ICU beds in India at USD 4925 annually. Bijlmakers et al. [62] costed unspecified laundry costs apportioned to surgery at USD 4541 and USD 10,646 per year for 278-bed and 325-bed public hospitals in Zambia. Cornelissen et al. [73] costed laundry for operating theaters in two district hospitals in Malawi at USD 933 and USD 2777 per year. Jain et al. [74] reported USD 6646 for building renovations and equipment to establish a central laundry facility serving a 200-bed surgical center in India at USD 2098 for one-year of staff costs.

Three studies reported unspecified laundry costs or aggregated laundry with other expenses. Kumar et al. [40] costed unspecified laundry expenses at USD 4.38 and 8.72 per inpatient bed-day for polytrauma and neurosurgery ICUs in India, respectively. Ranasinghe et al. [54] aggregated sterilization and laundry costs for hemodialysis units in five different hospitals in Sri Lanka ranging from USD 61 to 286 per month. Baechler and Ortiz [64] combined unspecified laundry and cleaning supplies at USD 207 annually for a rural health post in Chile.

### 3.8. Study Quality Assessment

**Figure 4:**
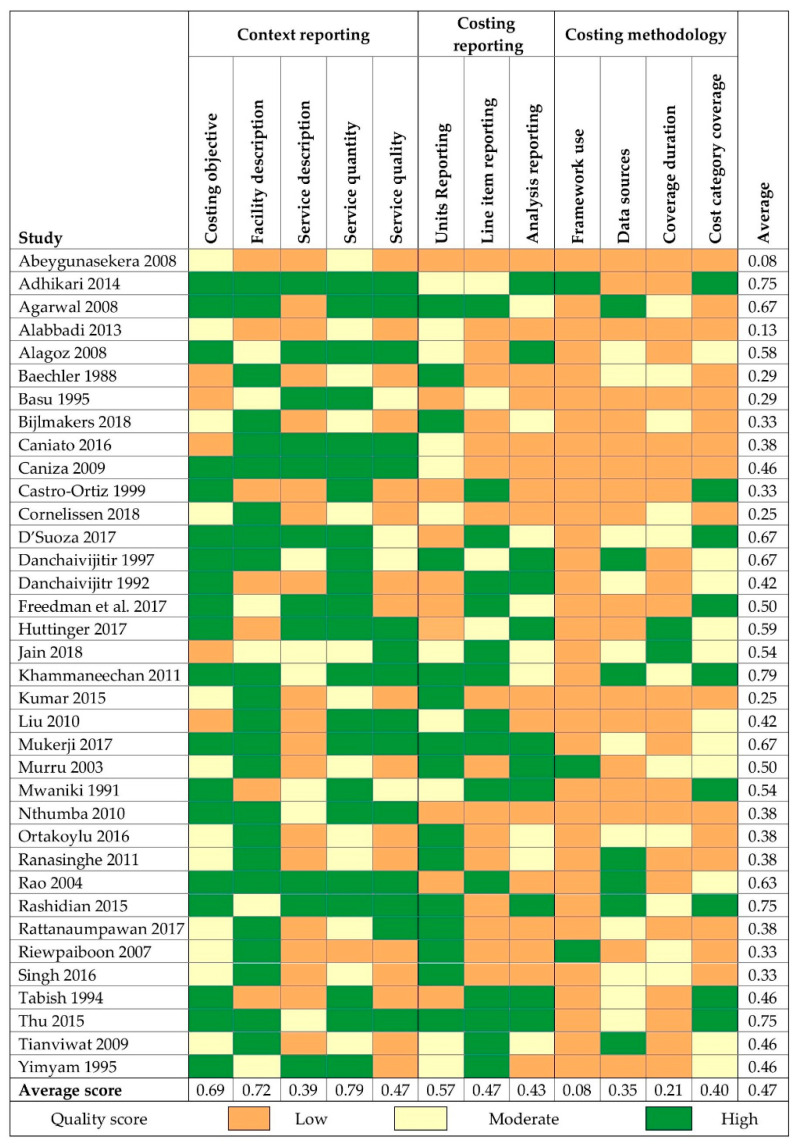
Quality assessment of studies reporting eligible costs data. For studies reporting multiple EHS, only the scores for the EHS with the lowest average quality ranking are shown. For a complete table of all quality scores by EHS, see Supplementary Information File 7. Average scores calculated as 0 for low, 0.5 for moderate, and 1 for high.

Most studies were rated as low quality for a majority of the 12 criteria evaluated (linear-weighted Cohen’s κ = 0.69 for inter-rater reliability). Where a study costed multiple EHS, Figure 4 depicts the results for the EHS with the lowest overall quality score, although scores across EHS were comparable in most cases. Average overall scores were highest for PPE, hygiene, and waste management. Lowest overall scores were for water, sanitation, and laundry. All quality scores for each EHS by study are included in Supplementary Information File 7.

Overall, reporting of study context scored highest. Most EHS reported a costing-related objective (88%) and provided at least some description of the HCF (76%). Descriptions of resource inputs necessary to provide each EHS were less common: 12 (24%) EHS described resource inputs for at least four of the cost categories included in Table 1 (excluding contracted services). A majority of EHS reported at least one indicator (47%) or direct measure (51%) of EHS quantity. Few studies provided any description of EHS quality across all included EHS (37%).

Reporting of costing methods was poor. For most studies, we judged that the detail reported on costing procedures was insufficient to replicate the analysis. The line items included in costs calculations were infrequently reported, and most studies included only a subset of costs required for EHS delivery (Figure 3). Most EHS (65%) included costs data for six months of expenses or less or did not report any data coverage dates. Framework use was reported for only three EHS (6%).

## 4. Discussion

### 4.1. Available Evidence

The purpose of this review was to evaluate the costs of establishing, operating, and maintaining EHS in HCFs of LMICs. We found little rigorous evidence. Of all EHS, cleaning was most commonly represented in the literature, followed by waste management and laundry. We found fewer than a dozen studies each costing water, sanitation, hygiene, and PPE, and no studies for lighting or vector control. Collection and aggregation of EHS with other non-EHS expenses was common, particularly for studies without an environmentally-focused costing objective, and as a result we excluded 29 studies.

Studies primarily divided into two disciplinary groups, reflecting different costing objectives. The first group comprised studies broadly within the field of healthcare economics and health policy, with the objective of understanding the costs of healthcare delivery or facility-wide operation. These studies typically employed top-down costing, which uses weighting criteria, such as floor area or patient volume, to allocate total costs for a particular system to individual units or services [75]. For example, Abeygunasekera et al. [53] allocated hospital-wide costs for various EHS to a urology unit using bed number as a weighting criterion. Top-down costing studies often included multiple EHS as single line item for overhead without further specification.

The second group comprised studies within the field of engineering, with the objective of understanding technology costs of systems required for EHS provision, most commonly waste management and sanitation. These studies employed bottom-up costing, which assesses the quantities and prices of individual resource inputs to estimate overall costs [75]. These studies typically collected and reported more detailed lifecycle costs but were also narrower in scope, considering costs of closed loop engineering systems that reflected only part of the broader EHS needs within a facility.

Reviews of costing approaches argue that neither top-down nor bottom-up costing should be considered a “gold standard” but rather reflect differing costing goals, and costing stakeholders should therefore consider the intended use of data to select an approach which is fit for purpose [25]. Top-down approaches may be used to evaluate long-term average costs, while bottom-up approaches provided a detailed snapshot of local costs and their variation [76]. Approaches are also limited by available records systems and data. Where EHS provision and purchasing is divided across multiple departments and centralized budgets do not exist, top-down costing is challenging. In contrast, bottom-up costing requires much more intensive data collection to document all resource inputs. This level of detail may be neither feasible nor necessary in all circumstances [25].

### 4.2. Cost Coverage

We found that costing specific activities or components of an EHS that are necessary but not sufficient for safe and adequate delivery was common, such as drinking water provision or waste transportation only. Overall, less than a quarter of EHS (n = 9) included at least four of the eight cost categories we considered, and a third of EHS (n = 17) provided no description of included expenses. Furthermore, within each cost category, we judged the line items included were likely insufficient to provide adequate EHS in most cases. Tangible goods in the form of consumables and capital hardware were most commonly costed. Capital maintenance, capital software, or recurrent training were costed by less than a quarter of studies. In some cases, omission of cost categories was explicitly recognized as a limitation, but most commonly these categories were simply never discussed. Omission of entire costs categories and expenses within categories suggests that existing evidence significantly underestimates the true costs of EHS provision.

Within the capital hardware category, construction costs were infrequently represented. Lack of evidence for construction, rehabilitation, or upgrades to EHS integrated into facilities where direct patient care was provided is concerning. Studies of EHS conditions worldwide indicate that, while HCFs may lack adequate, safe EHS, many are still providing EHS at some level [19]. In these cases, understanding costs of upgrading or rehabilitating existing infrastructure is more salient and aligns with the service ladder concept, in which infrastructure is built and EHS improved through incremental improvements [77,78].

We found that costs were not representative across the lifecycle of technologies for included studies. Most studies collected costs data for six months or less. While many EHS require infrastructure that can have substantial final disposal costs, we found no studies including expenses related to the disposal phase of the costing lifecycle. We found that EHS maintenance was rarely costed, either through omission, explicit exclusion, or aggregation with general building costs or other expenses. Labor and supervisory costs incurred during all phases of the costing lifecycle are similarly important but were infrequently reported.

While we did not find lifecycle costs holistically evaluated by any of the eligible studies yielded by our search, other studies have suggested that lifecycle costing is important to capture costs that may not be distributed evenly across a technology’s lifespan [79]. Collection of maintenance costs over just a portion of a technology’s lifespan may only partially reduce bias. Costs collected for only several months are unlikely to capture infrequent, high-cost repairs, and total maintenance costs are generally unevenly distributed across time, with more costs incurred as infrastructure ages [30,80]. Consideration of lifecycle costs is important particularly when phases are funded by different entities to ensure that no inappropriate trade-offs are made between low upfront but high overall lifecycle costs.

Selection of timeframes and included costs have broader implications for investing in cost-effective and sustainable technology [81]. For example, studies of reusable versus disposable products that account for the costs of cleaning and reuse but not disposal costs risk coming to biased cost-effectiveness estimates. For example, Danchaivijit et al. [66] and Yimyam et al. calculate costs of cleaning for reusable syringes but not waste management costs associated with disposable syringes. As a result, the existing evidence provides an incomplete picture of lifecycle costs and should be interpreted with caution.

### 4.3. Costing Methods and Reporting

We found costing methodology and reporting deficient in several ways. Methods for calculating total and unit costs were incompletely reported by 39 studies. Common omissions included data sources and methodology for calculating unit costs, discounting, and apportioning. Studies that included EHS costs under objectives to cost facility-wide operation or service provision rarely reported details of methodology for EHS-specific costing. Most studies either did not report data sources or used data sources that are subject to recall bias (n = 27, 53%). Recall bias is a well-documented phenomenon [82], though we found little discussion of the validity or limitations of data sources. Triangulation is a mixed-methods approach to combine qualitative information about quantitative data to assess validity and reduce possible bias [83], yet we found no studies triangulating between multiple data sources.

Price paid versus market price were infrequently distinguished and often inappropriately interchanged. Price paid represents the amount paid for a good or service, whereas market price represents the value of the resources used for EHS production. For example, the price paid may fail to capture costs of procurement and transportation, which can be substantial. We found that price paid was more commonly used. Our search yielded multiple studies that considered donated supplies and uncompensated staff labor to be “free” and omitted these expenses from cost calculations (see, e.g., [46,72]). Other reviews have also suggested that the use of budgets as a measure of costs may prove unreliable, given discrepancies between allocations, disbursements, and expenditures [84], and that to accurately value EHS, analyses should use market prices [75,84].

Finally, cost units were inconsistently or incompletely reported. The precise timeframe of costs data was not reported for 45 EHS. Furthermore, studies should report the dates during which expenses were incurred, rather than the dates of field visits to retrieve or analyze records, which we found were often reported but not meaningful for inflation adjustments. Foreign exchange rates were rarely reported, though currency conversions were common. We did not require reporting of costs in international dollars as a criterion for quality scoring, though inclusion of this information would facilitate comparison across studies. Reporting currency units and dates of expenses is critical to appropriately account for inflation to compare results across studies [75].

Reporting standards for costing studies exist [37,38] and should be more widely applied. Anderson et al. [25] propose a ten-step model for budgeting for EHS in HCFs that outlines key steps for costing, as well as assessing the EHS and HCF context, that may be applied to improve methodologic rigor of costing studies.

### 4.4. Context Reporting—Service Quantity and Quality

Reporting of facility characteristics, EHS quantity and EHS quality was poor. Facility descriptions were often limited to the type of facility and/or bed number. More detailed descriptions—such as number of outpatient procedures, deliveries, or inpatient bed-days—were rare. Furthermore, these indicators are likely insufficient to assess EHS demand. We found several studies to suggest that demand comprises fixed and variable costs components [55,58,61,85], and variable costs may not scale linearly with patient volume [61]. Fixed costs typically represent capital hardware and software investments, whereas operations and maintenance expenses are more elastic. Economies of scale may occur, as we found some evidence to suggest that larger facilities had lower per patient costs [54].

Direct measures of EHS demand may be more appropriate, though we found these rarely reported. Most were reported for waste management services using mass or volume of waste treated or produced. Some EHS lend themselves well to direct measurement through existing metering technologies, such as inflow of water or outflow of sewage, though we did not find these measures reported. Demand for PPE may be directly measured through the number of procedures performed or equipment used [71], though training demands for proper use may be more challenging to quantify directly. Hygiene and cleaning have a wide variety of inputs and less tangible outputs, which prove more changing to measure. Floor space was commonly used as a criterion to apportion cleaning but is likely a poor proxy for EHS demand, as cleaning frequency and sterilization needs vary widely by surface and device type and intensity of use [85]. Use of individual products, for example antiseptics [69], may have potential to serve as more accurate proxies, provided they correlate well with overall EHS demands.

Lack of well-defined and consistently applied measures of EHS quantity likely contributes to heterogeneity in reported unit costs. Costs were most commonly reported at facility-level in units of operating costs per month or year, with capital hardware and software costs—where included—either annualized over an assumed technology lifespan or reported as fixed start-up costs. Only eight studies reported unit costs in terms of EHS quantity indicators [40,46,48,52,58,61,70,72], and we were able to compare unit costs across studies for only two direct measures: cost per kilogram of healthcare waste treated [44,55,56] and cost per syringe recycled [66,67]. Costs presented as a percentage of total facility expenses may be more readily generalized but were only presented by one study [86]. Where costs were reported in terms of total facility-wide costs, lack of corresponding descriptions of the facility context and EHS quantity limited the ability to compare and generalize findings.

EHS quality was even less commonly reported. Few (n = 19) studies reported any description of EHS quality. Lack of quality reporting hinders the ability to meaningfully compare costs across studies and may reflect a paucity of well-established definitions and indicators. EHS quality needs will vary by the type of healthcare services provided within an HCF. For example, an ICU treating burn victims will have different water quality needs than an outpatient clinic providing vaccination services. For household settings, the WHO uses concentration of *Escherichia coli* as an indicator of water quality, with different risk levels defined by concentrations [87], but specifically excludes special needs settings such as HCFs. *E. coli* concentration may be an appropriate indicator for HCF settings but will require acceptable risk levels for various applications to be defined, and recommendations regarding the frequency of testing and compliance rates may require revision.

Cost-benefit or cost-effectiveness studies offer the potential to evaluate quality more rigorously and compare results across facilities. We found three studies analyzing cost-benefit [61] or cost-effectiveness [39,52]. Of the studies included in this review, we found provider willingness to pay for improved working conditions documented as a benefit [61], and other studies suggest patient satisfaction and care seeking as benefits [10]. Cost-benefit studies have been conducted for WaSH in non-HCF settings [88], but how benefits would be measured in HCFs is unclear. Prevention of HAIs may be a useful outcome for assessing cost-effectiveness that is applicable across all EHS [89,90] but is likely to prove challenging and resource intensive to measure.

### 4.5. Limitations of This Review

Our search strategy used databases that included both journal publications and grey literature. We selected databases that covered medical, public health, environmental health, business, and economics disciplines relevant to our topic and included some grey literature. However, given the number of studies that collected but did not report disaggregated costs findings, we suspect that a broader evidence base is available than is represented both in this review and the published literature overall. A small number of eligible studies may have been excluded during the second phase of machine learning where we manually screened only studies with relevance scores greater than 0.5, though this is unlikely as all studies from the search update included in the final analysis were identified in the first phase of machine learning, and manual screening in the second phase yielded no eligible studies.

Nonacademic databases may also contain additional relevant grey literature that is not included in this study. We attempted to apply our search methodology to online databases from USAID [91] and the World Bank [92] to capture additional grey literature. However, these databases did not support export of references with titles and abstracts. They were therefore not compatible with the software we used for reference management and search update screening, and screening without the assistance of these tools was not feasible. Unpublished project budgets, contracts, and reports used by HCFs or funding agencies for internal accounting purposes would be valuable evidence that are missing from this review.

Our search was restricted to studies conducted in LMICs. We excluded studies from high-income countries because many of the modalities of EHS provision in these countries are neither feasible nor appropriate for LMICs. For example, flush toilets connected to municipal sewerage treatment plants are the norm for urban hospitals in high-income countries but are not feasible for most rural clinics in LMICs. While some reviews have been done in high-income settings on the costs of standard precautions and infection prevention measures (see, e.g., [93]), information on the costs of infrastructure components of EHS such as water supply and sanitation was lacking. To our knowledge, this is the first systematic review to examine the costs of the broad suite of EHS necessary to ensure safe and functional HCFs in high-income countries or LMICs.

Our ability to compare findings across studies was poor. Descriptions of facility characteristics required to facilitate meaningful comparisons were often sparse, with little to no indication of the facility size, type of healthcare services provided, or EHS quantity needs. We relied on authors’ descriptions of facility types (e.g., “teaching hospital” versus “district hospital”) rather than a standard typology, which, to the best of our knowledge, does not exist. Included studies did not reliably report information that would be meaningful to contextualize and compare EHS delivery, such as healthcare services provided at an HCF or measures of EHS demand. Bed number was the most reported facility characteristic, but we found no evidence that this is a reliable indicator for EHS quantity or patient volume more broadly, as other factors such as occupancy rates and number of outpatients will also influence patient volume.

Heterogeneity in units of reported costs prevented comparison of findings across studies. Our protocol did not stipulate a meta-analysis, and the wide variation in EHS provision modalities, cost units used in reporting, and heterogeneity of facility types supports that a meta-analysis would have been neither appropriate nor feasible. While we standardized all currency to USD adjusted to inflation for October 2018, we otherwise reported costs in the units as originally described. We chose not to standardize the time horizon of included costs, for example by adjusting costs per any unit of time to a standard rate of costs per year. This decision was made in part to avoid bias from short time horizons included in most studies.

### 4.6. Implications and Future Research

Our search results suggest that the breadth and depth of data collected on EHS in HCFs exceeds that which we were able to disaggregate and report in this review. Lack of evidence is particularly challenging when constructing new facilities or undergoing substantial upgrades or rehabilitations, as funders and facility stakeholders are less likely to have prior data and internal records to appropriately plan and budget for these expenses and must rely on data from other sources.

In this review, we found that few studies on costs of EHS in HCFs exist, and those that do are poor quality. Building the evidence base will require additional efforts for data collection. Additionally, efforts to enhance access to existing EHS costs data which are not currently publicly available, particularly from non-journal and existing unpublished sources, would allow for better informed decision making. The rise of electronic health information management systems and online data sharing platforms offers potential to streamline collection and dissemination of EHS costs information. As costs data can be collected without ethical concerns related to human subjects research, open-access crowdsourced data sharing platforms, which have been applied in other areas of public health research [94], have the potential to build a wider body of evidence. Efforts to improve definitions and indicators for measurement of EHS quantity and quality in HCFs are necessary to contextualize costing studies. Indicator development will require additional research to identify robust and readily measurable indicators applicable to diverse contexts and facility types. Research to assess the relationships between patient volume, EHS demand, and costs would assist in developing reliable EHS quantity indicators. Efforts are also needed to identify robust measures of effectiveness and benefits that are comparable across EHS. Rigorous cost-effectiveness and benefit-cost studies are lacking but could assist policy makers in prioritizing improvements to EHS, particularly where insufficient budget is available to make improvements to all EHS in parallel or when rapid response is needed, such as during disease outbreaks.

Finally, this review indicates a need to develop theoretical frameworks describing the resource inputs and activities necessary to achieve adequate EHS provision. Use of costing frameworks that outline essential inputs and outputs for EHS delivery would help ensure that costing is comprehensive both across and within costs categories [25]. However, such frameworks are lacking. We found no standardized system for defining or classifying EHS needs in HCFs in any included studies, and to the best of our knowledge no such system exists. A variety of guidelines broadly outline environmental hygiene and infection prevention needs within HCFs (see, e.g., [17,86,93,95,96]), but descriptions of the specific inputs needed to meet these guidelines are insufficient to develop such costing frameworks.

Efforts to develop and apply frameworks and theories to conceptualize and measure EHS needs in HCFs should be a priority. This review considers only the existing empirical evidence on costs, but further research to develop such theories and frameworks would improve comprehensive costing and methodological rigor and could help policy makers and practitioners to identify and plan for EHS needs and advocate for adequate funding. Development of these tools is beyond the scope of this study, but we direct the reader towards related research advancing framework development (see, e.g., [25,26]).

## 5. Conclusions

Understanding costs is important to appropriately plan and budget for sustainable service delivery at facility-level, as well as to inform appropriate budgeting at the health system level to ensure that HCFs have adequate funding for safe and functional care delivery. However, existing literature offers few representative and generalizable findings about the costs of EHS delivery in HCFs. Overall, we found a paucity of studies, tools, and frameworks to guide costing specifically of EHS in HCFs but a wide range of approaches from other disciplines. This diversity of costing approaches and lack of focused tools has contributed to inconsistent reporting and low-quality evidence. Literature from healthcare disciplines offers the necessary breadth for holistic EHS delivery and contextualizing EHS quantity and quality needs, while literature from engineering disciplines offers the necessary depth for accurate and representative costing and the understanding of long-term lifecycle costs of infrastructure. Rigorous costing of EHS in HCFs will likely ultimately require a blend of multiple disciplinary approaches. More wide-spread application of indicators and methods designed specifically to measure and cost EHS in HCFs has the potential to improve rigor and generalizability of findings.

## Figures and Tables

**Table 1:** Definitions of costs categories. Adapted from Anderson et al. [25].

Cost Category	Definition
Capital hardware	Infrastructure or equipment purchases required to establish services or implement changes to service delivery method, which are not consumed during normal service operation
Capital maintenance	Expenses required to repair, rehabilitate, or otherwise maintain functionality of capital hardware, including labor costs required for these purposes
Capital software	Planning, procurement, and initial training costs associated with establishing new services or implementing changes to service delivery method
Recurrent training	Training required to ensure proper ongoing service provision regardless of changes to service delivery
Consumables	Products and supplies that are consumed during normal operation
Personnel	Labor costs associated with normal operation of a service, including staff benefits
Direct support	Expenses required to supervise and monitor service provision to ensure safety and sustainability that support but do not have direct service outputs, such as auditing or developing management plans
Financing	Loan interest and other fees associated with service financing
Contracted services	Fees paid to external providers to perform all or part of normal service operation, including multiple other cost categories, where expenses cannot be accurately disaggregated into categories above; where fees fall solely within another cost category described above, expenses should be included therein

## Data Availability

No new data were created or analyzed in this study. Data sharing is not applicable to this article.

## Supplementary Materials

The following are available online at https://www.mdpi.com/1660-4601/18/2/817/s1, Supplementary Information File S1: PRISMA checklist, Supplementary Information File S2: Review protocol, Supplementary Information File S3: Search details, Supplementary Information File S4: Extraction form and tables, Supplementary Information File S5: Quality scoring tool, Supplementary Information File S6: Listing of selected excluded studies, Supplementary Information File S7: Quality scoring results.
