# Supplementary material for: Safe Healthcare Facilities: A Systematic Review on the Costs of Establishing and Maintaining Environmental Health in Facilities in Low- and Middle-Income Countries
Source: Int J Environ Res Public Health. 2021 Jan 19;18(2):817. doi: 10.3390/ijerph18020817 (PMC7833392; doi:10.3390/ijerph18020817)
Supplement: Supplementary file 1 [file ijerph-18-00817-s001.zip › Supplements/Supplementary Information File 2 - Protocol - EHS in HCF systematic review.docx]

Protocol

The costs of establishing and maintaining environmental health services in healthcare facilities in low- and middle-income countries: a systematic review

## Authors: Darcy M. Anderson^1*^, Ryan Cronk^1^, Jamie Bartram^1^

^1^The Water Institute, University of North Carolina at Chapel Hill, Chapel Hill, North Carolina, United States of America

* Corresponding author: [darcy.anderson@unc.edu](mailto:darcy.anderson@unc.edu)

The Water Institute at UNC

Gillings School of Global Public Health

The University of North Carolina at Chapel Hill

Rosenau Hall, CB #7431

135 Dauer Drive, Chapel Hill, NC 27599-7431

Last update: 5 June, 2019

# Background

Safe and hygienic environmental conditions are critical for providing quality patient care and preventing healthcare-acquired infections (HCAIs). The global burden of HCAIs is significant, with reviews suggesting that infection rates may be as high as 15% of all hospitalized patients in low- and middle-income countries [1,2]. Estimates suggest that approximately 6% of patients with an HCAI die from related causes in high-income settings, and rates may be higher in low-resource settings where appropriate treatment is more challenging [3].

Environmental contamination in the healthcare environment is a major contributor to the burden of HCAIs. An estimated 40-60% of HCAIs are transmitted on the hands of healthcare personnel, and an additional 20% are attributable to environmental contamination [4]. Provision of basic environmental health services (EHS) in healthcare facilities (HCFs) is considered critical for safe care delivery and [5,6] and may also improve patient satisfaction and reduce barriers to care seeking behavior [7].

Under the Sustainable Development Goals, Goal 6 calls for universal access to water, sanitation, and hygiene (WASH), where ‘universal’ is defined as all settings including HCFs, as interpreted by the World Health Organization (WHO) and United Nations Children's Fund Joint Monitoring Program for Water Supply, Sanitation and Hygiene (JMP), which has the mandate for monitoring Goal 6 [8]. Furthermore, HCFs bear additional EHS requirements beyond WASH for functional service delivery and prevention of HCAIs [9]. In response, the JMP has under development indicators for monitoring healthcare settings that include hygiene availability to healthcare providers at points of care and medical waste disposal [10]. While not included under the JMP monitoring mandate, the WHO standards for essential EHS in HCFs also include food hygiene; laundry; energy and lighting; ventilation, heating and cooling; and vector control [11].

HCFs have been identified as an area of urgent need in WASH [12]. An estimated 50% of HCFs in low- and middle-income countries lack piped water; 33% lack improved sanitation; 39% lack handwashing soap; 39% lack adequate infectious waste disposal; and 73% lack sterilization equipment [13]. Progress towards provision of other EHS not monitored under the JMP mandate is similarly lacking. Approximately 26% of HCFs in sub-Saharan Africa lack access to a reliable electricity supply [14].

Lack of understanding of costs required for EHS provision have been identified as a barrier to informed budgeting and progress towards achieving universal access [9]. Of the countries included in the WHO’s Global Analysis and Assessment of Sanitation and Drinking-Water, approximately half have developed and costed a national implementation policy for hygiene promotion (52%), sanitation and drinking water (58%), and infection prevention and control (57%). Only 22% have a financing plan in place for WASH in HCF that is consistently implemented [15].

Paucity of costs data has led to calls for greater collection and application of evidence to inform investment in EHS in HCF [9,16], yet a corresponding response from the research and allied communities has been lacking. The goal of this systematic review is to determine the costs of providing EHS in HCFs. We will evaluate the costs of service provision in eight focus areas identified in the WHO’s *Essential environmental health standards in health care* [11]: water, sanitation, hygiene, waste management, surface and medical device cleaning and sterilization (hereafter referred to as “cleaning”), personal protective equipment (PPE), laundry lighting, and vector control.

# Objectives

The specific objectives of this review are:

- To document the expenditures associated with establishing and maintaining EHS in HCFs
- To estimate the costs of establishing and maintaining EHS in HCFs
- To assess gaps in available evidence and outline recommendations for improving future costing

This review will be restricted to studies conducted in low- and middle-income countries.

# Methods

## Reporting

We will adhere to the Preferred Reporting Items for Systematic Reviews (PRISMA) statement [17].

## Search strategy

We will systematically search PubMed, Scopus, Ebsco (Global Health and Business Source Premier), Web of Science, and Proquest Theses and Dissertations Global databases for relevant journal articles. Databases will be searched using Boolean operators for at least one term from four clusters of terms related to 1) EHS, 2) HCFs, 3) costing and financial analysis, and 4) low- and middle-income countries. EHS terms will be designed to capture services related to the nine focus areas identified in the WHO guidelines (water, sanitation, hygiene, waste management, cleaning, PPE, laundry, lighting, and vector control) [11]. Healthcare facility terms will include a variety of facility settings and sizes, ranging from large hospitals to rural posts and dispensaries, as well as facilities specializing in dental and maternal health services. Search terms are provided in Box 1.

We will conduct all searches in English. Ebsco, Scopus, and Web of Science will be searched using a restriction for terms included in titles, abstracts, and keywords only. Proquest Theses and Dissertations Global will be searched for all fields except full-text. PubMed will be searched using the native search function. All databases will be searched from inception. References will be managed using Covidence software for systematic reviews (Veritas Health Innovation, Melbourne, Australia).

## Eligibility

We will include English, French, and Spanish language studies, regardless of publication status. Studies will eligible for inclusion if they:

- Evaluate the expenditures or overall costs associated with providing an EHS in an HCF in a low- or middle-income country, and
- Attempt to holistically evaluate all expenditures and/or costs associated with providing a particular service at a facility or sub-facility level, such as within a department or unit

We will consider both studies that evaluate “hardware” (e.g. physical infrastructure, consumable products, and supplies) and “software” (e.g. hygiene promotion, IPC training). Both intervention studies reporting data on delivering new or upgraded EHS and observational studies reporting on existing facilities will be eligible.

We define a healthcare facility as any permanent institution with the primary purpose of delivering medical services. We will include studies conducted within specialized units, so long as environmental costs were evaluated for all care provided within the unit. Studies of healthcare delivered in institutions whose primary purpose is non-medical, such as schools or residential group homes, will be excluded. We will also exclude non-permanent facilities, such as temporary facilities established for vaccination campaigns or mobile clinics. We will also exclude studies that evaluate procedure-specific costs for only a subset of care provided.

Box 1: Search terms

$ indicates “wild card” characters, which can be represented by any letter or no letter

* indicates truncations, for which any characters may follow the asterisk

**Environmental health services**

standard precautions OR infection control OR infection prevention OR IPC OR asepsis OR aseptic OR sterile OR sterility OR sterilization OR sterilisation OR personal protective equipment OR PPE OR splash protection OR respiratory protection OR mask$ OR glove$ OR gown$ OR scrubs OR goggles OR eye protection OR face shield$ OR respirator$ OR Sharps OR needle$ OR syringe$ OR Water OR Sanitation OR Sanitary OR Plumbing OR Sewage OR Sewer* OR latrine$ OR toilet$ OR hygiene OR Hygienic OR Shower$ OR Soap$ OR detergent$ OR Handwashing OR hand washing OR hand hygiene OR Laundry OR bedsheet$ OR bedding OR linen$ OR Waste$ OR Landfill$ OR Dump$ OR Drainage OR Wastewater OR waste water OR disposal OR lighting OR light$ OR vector control OR Vectors OR pest$ OR rodent$ OR rat$ OR insect$ OR vermin OR infest* OR fly OR flies OR mosquito OR mosquitoes OR Surface$ OR fomite$ OR Chlorine OR Disinfect* OR Cleaners OR cleaning OR cleanliness OR janitor* OR housekeep*

**Healthcare facilities**

healthcare OR “health care” OR hospital$ OR clinic$ OR “health facilit*” OR “health center$” OR healthcenter$ OR healthcentre$ OR “health centre$” OR “health post$” OR healthpost$ OR “health setting*” OR “medical facilit*” OR “medical center$” OR “medical centre$” OR “medical post$” OR “medical setting*” OR “delivery facilit*” OR “delivery center$” OR “delivery centre$” OR “delivery clinic$” OR “birth facilit*” OR “birth center$” OR “birth centre$” OR “birth clinic$” OR “matern* facilit*” OR “matern* center$” OR “matern* centre$” OR “matern* clinic$” OR “dental facilit*” OR “dental center$” OR “dental centre$” OR “dental clinic$” OR dispensary OR dispensaries

**Costing**

Cost$ OR costing OR economic OR budget* OR Price$ OR pricing OR fee$ OR tariff$ OR loan$ OR subsidy OR subsidies OR finance$ OR financial OR financing OR fund$ OR funding OR pay OR payment$ OR investment$ OR investing OR capital OR money OR monies OR expense$ OR accounting

**Low- and middle-income countries**

Afghanistan OR Algeria OR Angola OR Anguilla OR Antigua OR Barbuda OR Argentina OR Armenia OR Armenian OR Aruba OR Azerbaijan OR Bahamas OR Bahrain OR Bangladesh OR Barbados OR Benin OR Byelarus OR Byelorussian OR Belarus OR Belorussian OR Belorussia OR Belize OR Bhutan OR Bolivia OR Botswana OR Brazil OR Brunei OR “Burkina Faso” OR “Burkina Fasso” OR “Upper Volta” OR Burundi OR Urundi OR Cambodia OR “Khmer Republic” OR Kampuchea OR Cameroon OR Cameroons OR Cameron OR Camerons OR “Cape Verde” OR “Cayman Islands” OR “Central African Republic” OR Chad OR Chile OR China OR Colombia OR Comoros OR “Comoro Islands” OR Comores OR Mayotte OR Congo OR Zaire OR “Cook Islands” OR “Costa Rica” OR “Cote d'Ivoire” OR “Ivory Coast” OR Croatia OR Cuba OR Cyprus OR Djibouti OR “French Somaliland” OR Dominica OR “Dominican Republic” OR “East Timor” OR “East Timur” OR “Timor Leste” OR Ecuador OR Egypt OR “United Arab Republic” OR “El Salvador” OR Eritrea OR Ethiopia OR “Falkland Islands” OR “Las Malvinas” OR Fiji OR Gabon OR “Gabonese Republic” OR Gambia OR Gaza OR “Georgia Republic” OR “Georgian Republic” OR Ghana OR “Gold Coast” OR Greece OR Grenada OR Guatemala OR Guinea OR Guam OR Guadeloupe OR Guiana OR Guyana OR Haiti OR Honduras OR “Hong Kong” OR India OR Maldives OR Indonesia OR Iran OR Iraq OR Jamaica OR Jordan OR Kazakhstan OR Kazakh OR Kenya OR Kiribati OR Korea OR Kosovo OR Kuwait OR Kyrgyzstan OR Kirghizia OR “Kyrgyz Republic” OR Kirghiz OR Kirgizstan OR “Lao PDR” OR Laos OR Lebanon OR Lesotho OR Basutoland OR Liberia OR Libya OR Macau OR Madagascar OR “Malagasy Republic” OR Maldives OR Malaysia OR Malaya OR Malay OR Sabah OR Sarawak OR Malawi OR Nyasaland OR Mali OR Malta OR “Marshall Islands” OR Martinique OR Mauritania OR Mauritius OR “Agalega Islands” OR Mexico OR Micronesia OR “Middle East” OR Mongolia OR Montserrat OR Morocco OR Ifni OR Mozambique OR Myanmar OR Myanma OR Burma OR Namibia OR Nauru OR Nepal OR Niui OR “Netherlands Antilles” OR “New Caledonia” OR Nicaragua OR Niger OR Nigeria OR “Northern Mariana Islands” OR Oman OR Mayotte OR Muscat OR Pakistan OR Palau OR Palestine OR Panama OR Paraguay OR Peru OR Philippines OR Philipines OR Phillipines OR Phillippines OR Polynesia OR “Puerto Rico” OR Qatar OR Reunion OR Rwanda OR Ruanda OR “Saint Kitts” OR “St Kitts” OR Nevis OR “Saint Lucia” OR “St Lucia” OR “Saint Vincent” OR “St Vincent” OR Grenadines OR Samoa OR “Samoan Islands” OR “Navigator Island” OR “Navigator Islands” OR “Sao Tome” OR “Saudi Arabia” OR Senegal OR Serbia OR Montenegro OR Seychelles OR “Sierra Leone” OR Singapore OR “Sri Lanka” OR Ceylon OR “Solomon Islands” OR Somalia OR “South Africa” OR Sudan OR Suriname OR Surinam OR Swaziland OR Syria OR Tajikistan OR Tadzhikistan OR Tadjikistan OR Tadzhik OR Tanzania OR Thailand OR Togo OR “Togolese Republic” OR Tokelau OR Tonga OR Trinidad OR Tobago OR Tunisia OR Turkey OR Turkmenistan OR Turkmen OR “Turks Caicos” OR “Turks and Caicos” OR Tuvalu OR Uganda OR “United Arab Emirates” OR Uruguay OR Uzbekistan OR Uzbek OR Vanuatu OR “New Hebrides” OR Venezuela OR Vietnam OR “Viet Nam” OR “Virgin Islands” OR “West Bank” OR Yemen OR Yugoslavia OR Zambia OR Zimbabwe

## Data extraction

For each unique study captured by our search, two reviewers will independently screen titles and abstracts for eligibility. Subsequent full-text review will also be conducted by two independent reviewers. For eligible studies, data will be extracted by two independent reviewers using Excel-based extraction form. Pilot testing will entail application of the form to three included studies by all reviewers, followed by comparison of extraction results to identify discrepancies in application or understanding of the form across reviewers. Extracted data will include: facility type, location, and indicators of patient volume; service description and included costs; costing methodology; and main findings of cost calculations. Where EHS data are aggregated with other costs, we will contact authors to request disaggregated data. All differences in screening and extraction results will be resolved by discussion.

## Data synthesis

For studies that include multiple EHS, we will extract each service separately. Costs will be coded as capital hardware, capital software, capital maintenance, consumables, personnel, recurrent training, direct support, financing, and contracted services, adapting categories from tools to cost WASH in community settings [18]. Definitions for each category are provided in Table 1.

All costs will be first converted into United States dollars using an online currency converter (<https://www.oanda.com/us-en/>) based on the historical foreign exchange rate at the date of data collection, and then adjusted for inflation to a fixed date following completion of extraction using a consumer price inflation calculator (<https://www.bls.gov/data/inflation_calculator.htm>). We will use the conversion rate for the median date of the data coverage period. For studies reporting years only, we will use January 1. For studies reporting no dates of data coverage, we will use the publication date. Where costs data are reported in multiple currencies, we will preferentially extract data in US dollars, where available.

Due to high expected heterogeneity in technologies and approaches used for EHS provision, costing methodologies, and specific cost components included, we do not anticipate that a meta-analysis will be feasible or appropriate for reporting cost data.

Table 1. Definitions of expenses included under costs categories

| **Cost category** | **Definition** |
| --- | --- |
| Capital hardware | Infrastructure or equipment purchases required to establish services or implement changes to service delivery method, which are not consumed during normal service operation |
| Capital maintenance | Expenses required to repair, rehabilitate, or otherwise maintain functionality of capital hardware, including labor costs required for these purposes |
| Capital software | Planning, procurement, and initial training costs associated with establishing new services or implementing changes to service delivery method |
| Recurrent training | Training required to ensure proper ongoing service provision regardless of changes to service delivery |
| Consumables | Products and supplies that are consumed during normal operation |
| Personnel | Labor costs associated with normal operation of a service, including staff benefits |
| Direct support | Expenses required to supervise and monitor service provision to ensure safety and sustainability that support but do not have direct service outputs, such as auditing or developing management plans |
| Financing | Loan interest and other fees associated with service financing |
| Contracted services | Fees paid to external providers to perform all or part of normal service operation, including multiple other cost categories, where expenses cannot be accurately disaggregated into categories above; where fees fall solely within another cost category described above, expenses should be included therein |

# Assessing study quality

We will assess study quality using a tool that we developed informed by guidelines for reporting healthcare economic studies [19-21]. Studies will be independently rated by two reviewers as high (+1 point), moderate (+0.5 points), or low (0 points) quality for 12 items related to context reporting (costing objective, facility description, service quantity indicators, service quality indicators), costing reporting (units reporting, line item reporting, analysis reporting), and costing methodology (framework use, data sources, coverage duration, and cost category coverage). Where reviewers disagree, disputes will be resolved by discussion. Each EHS described by a study will be scored separately. The scoring tool is included in Appendix A.

# Acknowledgements

This work is supported by a grant from the Wallace Genetic Foundation. DA is supported by the Royster Society of Fellows at the University of North Carolina at Chapel Hill. The authors declare no conflicts of interest.

# References

1. Allegranzi B, Bagheri Nejad S, Combescure C, Graafmans W, Attar H, et al. (2011) Burden of endemic health-care-associated infection in developing countries: systematic review and meta-analysis. Lancet 377: 228-241.

2. Bagheri Nejad S, Allegranzi B, Syed SB, Ellis B, Pittet D (2011) Health-care-associated infection in Africa: a systematic review. Bull World Health Organ 89: 757-765.

3. Klevens RM, Edwards JR, Richards CL, Jr., Horan TC, Gaynes RP, et al. (2007) Estimating health care-associated infections and deaths in U.S. hospitals, 2002. Public health reports (Washington, DC : 1974) 122: 160-166.

4. Weinstein RA (1991) Epidemiology and control of nosocomial infections in adult intensive care units. The American journal of medicine 91: S179-S184.

5. Pittet D, Allegranzi B, Storr J, Donaldson L (2006) ‘Clean Care is Safer Care’: the Global Patient Safety Challenge 2005–2006. International Journal of Infectious Diseases 10: 419-424.

6. Ducel G, Fabry J, Nicolle L (2002) Prevention of hospital-acquired infections. Geneva: World Health Organization.

7. Bouzid M, Cumming O, Hunter PR (2018) What is the impact of water sanitation and hygiene in healthcare facilities on care seeking behaviour and patient satisfaction? A systematic review of the evidence from low-income and middle-income countries. BMJ Glob Health 3: e000648.

8. United Nations General Assembly (2015) Transforming our World: the 2030 Agenda for Sutstainable Development. New York, New York: United Nations.

9. D'Mello-Guyett L, Cumming O (2016) Water, sanitation and hygiene in health care facilities: global strategy, burden of disease, and evidence and action priorities. London.

10. WHO/UNICEF (2016) Expert Group Meeting on Monitoring WASH in Health Care Facilities in the Sustainable Development Goals. Geneva.

11. Adams J, Bartram J, Chartier Y (2008) Essential environmental health standards in health care Geneva: World Health Organization.

12. WHO (2013) Opening remarks: Budapest Water Summit. Budapest.

13. Cronk R, Bartram J (2018) Environmental conditions in health care facilities in low- and middle-income countries: Coverage and inequalities. International Journal of Hygiene and Environmental Health.

14. Adair-Rohani H, Zukor K, Bonjour S, Wilburn S, Kuesel AC, et al. (2013) Limited electricity access in health facilities of sub-Saharan Africa: a systematic review of data on electricity access, sources, and reliability. Glob Health Sci Pract 1: 249-261.

15. WHO (2017) UN-Water global analysis and assessment of sanitation and drinking-water (GLAAS) 2017 report: financing universal water, sanitation and hygiene under the sustainable development goals. Geneva: WHO.

16. WHO and UNICEF (2019) Water, sanitation and hygiene in health care facilities: practical steps to achieve universal access to quality care. Geneva: WHO. 9241515511 9241515511.

17. Moher D, Liberati A, Tetzlaff J, Altman DG (2009) Preferred reporting items for systematic reviews and meta-analyses: the PRISMA statement. PLoS Med 6: e1000097.

18. Schwelltzer RW, Grayson C, Lockwood H (2014) WASHCost Tool.

19. Walker DG, Wilson RF, Sharma R, Bridges J, Niessen L, et al. (2012) Best Practices for Conducting Economic Evaluations in Health Care: A Systematic Review of Quality Assessment Tools. Rockville (MD): Agency for Healthcare Research and Quality (US).

20. Husereau D, Drummond M, Petrou S, Carswell C, Moher D, et al. (2013) Consolidated Health Economic Evaluation Reporting Standards (CHEERS) statement. BMJ : British Medical Journal 346: f1049.

21. Drummond MF, Jefferson TO (1996) Guidelines for authors and peer reviewers of economic submissions to the British Medical Journal. British Medical Journal 313.

# Appendix A: Quality scoring tool

## Definitions

- **Costing framework:** a tool that describes and categorizes expenditures required for service provision, and that may be used to identify expected costs
- **Indicators of service quantity**: an indicator of patient volume or other facility characteristics that may serve as a proxy for service demand
- **Direct measures of service quantity**: a direct measure of a service input or output

|  |  | **High quality (1 point)** | **Moderate quality (0.5 points)** | **Low quality (0.0 points)** |
| --- | --- | --- | --- | --- |
| **Context reporting** | Costing objective | Goal, objective, and/or research questions related to costing the specific EHS are reported | Goal, objective, and/or research questions related to costing are reported but are not EHS specific | Overall research goal is stated but costing objectives are not reported or are unclear OR no stated goals, objectives, or research questions |
|  | Facility description | All of the following are reported: facility type (e.g. teaching hospital, health post) and geographic location | At least one of the following are reported: facility type (e.g. teaching hospital, health post) or geographic location | Neither facility type nor geographic location are reported |
|  | Service description | Resources necessary to provide the service are described for each of the following categories: infrastructure and equipment, personnel, consumable products, trainings | Resources necessary to provide the service are reported for at least two of the following categories: infrastructure and equipment, personnel, consumable products, trainings | Resources necessary to provide the service are reported for less than two of the following categories: infrastructure and equipment, personnel consumable products, trainings |
|  | Service quantity indicators | At least one direct measure of the service quantity is reported | At least one indicator of service quantity is reported | No direct measures or indicators of service quantity are reported |
|  | Service quality indicators | At least one quantitative indicator of service quality (i.e. a SMART indicator with all components defined, either within the paper or by an external organization or government body) is reported OR a cost-effectiveness or cost-benefit analysis are conducted | Service quality is narratively described without using quantitative indicators | Service quality is not reported or claimed without substantiation |
| **Costing reporting** | Units reporting | All relevant cost units are reported, including time period for operation costs; currency; year of costs data reported; foreign exchange rates; and inflation adjustments, where relevant | Currency and units for time-bound costs (where relevant) are reported, but other relevant information, such as year of costs data, foreign exchange rate, or inflation adjustment, are not reported or unclear | Currency units are not explicitly stated or ambiguous (e.g. “dollars” or $ versus USD or CAD) |
|  | Line item reporting | All line items included in cost calculations are reported | Categories of expenses included in costs are reported but not specific line items | No description of line items included in costs, or only a subset of line items are listed as examples but not representative of all items included |
|  | Analysis reporting | Methodology for calculating all costs is reported in full, including methodologies for annualizing capital costs, discounting, apportioning, and calculating unit costs | Methodologies for calculating costs are broadly reported but specific details (e.g. rate use for discounting or criteria used for apportioning) are not reported or methodologies for only a subset of steps are reported | Methodologies for calculating costs are not reported or unclear |
| **Costing methodology** | Framework use | A costing framework was used *a priori* to structure data collection | A costing framework was used but not selected *a priori* to structure data collection or timing of framework use was not specified as a priori | No costing framework was used |
|  | Data sources  [data sources refers specifically for costing the EHS; a study may use multiple data sources for non-EHS cost components but these are not relevant to quality scoring] | Cost data were collected using multiple methods, including at least one method that does not rely on participant recall (e.g. records review, structured observation); findings were compared across methods and/or collaboratively assessed by facility staff for validity | Cost data collection method uses a single source for each calculation input and does not rely on participant recall | Data collection relies on recall only or data source not reported/unclear |
|  | Coverage duration | Costs data covering > 12 months of expenses | Costs data cover > 6 to ≤ 12 months of expenses | Costs data cover ≤ 6 months of expenses or no coverage dates are reported |
|  | Cost category coverage | Costs include expenses from at least 4 of the following categories: direct support, capital hardware, capital software, capital maintenance, consumable products, personnel, training, financing | Costs include expenses from at least 3 of the following categories: direct support, capital hardware, capital software, capital maintenance, consumable products, personnel, training, financing | Costs include expenses from fewer than 3 of the following categories: direct support, capital hardware, capital software, capital maintenance, consumable products, personnel, training, financing |
