# Supplementary material for: Safe Healthcare Facilities: A Systematic Review on the Costs of Establishing and Maintaining Environmental Health in Facilities in Low- and Middle-Income Countries
Source: Int J Environ Res Public Health. 2021 Jan 19;18(2):817. doi: 10.3390/ijerph18020817 (PMC7833392; doi:10.3390/ijerph18020817)
Supplement: Supplementary file 1 [file ijerph-18-00817-s001.zip › Supplements/Supplementary Information File 3 - Search details - EHS in HCF systematic review.docx]

## Search details

$ = wildcard, can be any single character or no character

* = truncation, any characters can follow, or no characters, to form a single word

**ENVIRONMENTAL CONDITIONS**

“standard precautions” OR “infection control” OR “infection prevention” OR IPC OR asepsis OR aseptic OR sterile OR sterility OR sterilization OR sterilisation OR “personal protective equipment” OR PPE OR “splash protection” OR “respiratory protection” OR mask$ OR glove$ OR gown$ OR scrubs OR goggles OR “eye protection” OR “face shield$” OR respirator$ OR Sharps OR needle$ OR syringe$ OR Water OR Sanitation OR Sanitary OR Plumbing OR Sewage OR Sewer* OR Latrine$ OR Toilet$ OR Hygiene OR Hygienic OR Shower$ OR Soap$ OR detergent$ OR Handwashing OR “hand washing” OR “hand hygiene” OR Laundry OR bedsheet$ OR bedding OR linen$ OR Waste$ OR Landfill$ OR Dump$ OR Drainage OR Wastewater OR “waste water” OR disposal OR lighting OR light$ OR “vector control” OR Vectors OR pest$ OR rodent$ OR rat$ OR insect$ OR vermin OR infest* OR fly OR flies OR mosquito OR mosquitoes OR Surface$ OR fomite$ OR Chlorine OR Disinfect* OR Cleaners OR cleaning OR cleanliness OR janitor* OR housekeep*

**HEALTHCARE FACILITIES**

healthcare OR “health care” OR hospital$ OR clinic$ OR “health facilit*” OR “health center$” OR healthcenter$ OR healthcentre$ OR “health centre$” OR “health post$” OR healthpost$ OR “health setting*” OR “medical facilit*” OR “medical center$” OR “medical centre$” OR “medical post$” OR “medical setting*” OR “delivery facilit*” OR “delivery center$” OR “delivery centre$” OR “delivery clinic$” OR “birth facilit*” OR “birth center$” OR “birth centre$” OR “birth clinic$” OR “matern* facilit*” OR “matern* center$” OR “matern* centre$” OR “matern* clinic$” OR “dental facilit*” OR “dental center$” OR “dental centre$” OR “dental clinic$” OR dispensary OR dispensaries

**COSTING**

Cost$ OR costing OR economic OR budget* OR Price$ OR pricing OR fee$ OR tariff$ OR loan$ OR subsidy OR subsidies OR finance$ OR financial OR financing OR fund$ OR funding OR pay OR payment$ OR investment$ OR investing OR capital OR money OR monies OR expense$ OR accounting

**LMICs**

Afghanistan OR Algeria OR Angola OR Anguilla OR Antigua OR Barbuda OR Argentina OR Armenia OR Armenian OR Aruba OR Azerbaijan OR Bahamas OR Bahrain OR Bangladesh OR Barbados OR Benin OR Byelarus OR Byelorussian OR Belarus OR Belorussian OR Belorussia OR Belize OR Bhutan OR Bolivia OR Botswana OR Brazil OR Brunei OR “Burkina Faso” OR “Burkina Fasso” OR “Upper Volta” OR Burundi OR Urundi OR Cambodia OR “Khmer Republic” OR Kampuchea OR Cameroon OR Cameroons OR Cameron OR Camerons OR “Cape Verde” OR “Cayman Islands” OR “Central African Republic” OR Chad OR Chile OR China OR Colombia OR Comoros OR “Comoro Islands” OR Comores OR Mayotte OR Congo OR Zaire OR “Cook Islands” OR “Costa Rica” OR “Cote d'Ivoire” OR “Ivory Coast” OR Croatia OR Cuba OR Cyprus OR Djibouti OR “French Somaliland” OR Dominica OR “Dominican Republic” OR “East Timor” OR “East Timur” OR “Timor Leste” OR Ecuador OR Egypt OR “United Arab Republic” OR “El Salvador” OR Eritrea OR Ethiopia OR “Falkland Islands” OR “Las Malvinas” OR Fiji OR Gabon OR “Gabonese Republic” OR Gambia OR Gaza OR “Georgia Republic” OR “Georgian Republic” OR Ghana OR “Gold Coast” OR Greece OR Grenada OR Guatemala OR Guinea OR Guam OR Guadeloupe OR Guiana OR Guyana OR Haiti OR Honduras OR “Hong Kong” OR India OR Maldives OR Indonesia OR Iran OR Iraq OR Jamaica OR Jordan OR Kazakhstan OR Kazakh OR Kenya OR Kiribati OR Korea OR Kosovo OR Kuwait OR Kyrgyzstan OR Kirghizia OR “Kyrgyz Republic” OR Kirghiz OR Kirgizstan OR “Lao PDR” OR Laos OR Lebanon OR Lesotho OR Basutoland OR Liberia OR Libya OR Macau OR Madagascar OR “Malagasy Republic” OR Maldives OR Malaysia OR Malaya OR Malay OR Sabah OR Sarawak OR Malawi OR Nyasaland OR Mali OR Malta OR “Marshall Islands” OR Martinique OR Mauritania OR Mauritius OR “Agalega Islands” OR Mexico OR Micronesia OR “Middle East” OR Mongolia OR Montserrat OR Morocco OR Ifni OR Mozambique OR Myanmar OR Myanma OR Burma OR Namibia OR Nauru OR Nepal OR Niui OR “Netherlands Antilles” OR “New Caledonia” OR Nicaragua OR Niger OR Nigeria OR “Northern Mariana Islands” OR Oman OR Mayotte OR Muscat OR Pakistan OR Palau OR Palestine OR Panama OR Paraguay OR Peru OR Philippines OR Philipines OR Phillipines OR Phillippines OR Polynesia OR “Puerto Rico” OR Qatar OR Reunion OR Rwanda OR Ruanda OR “Saint Kitts” OR “St Kitts” OR Nevis OR “Saint Lucia” OR “St Lucia” OR “Saint Vincent” OR “St Vincent” OR Grenadines OR Samoa OR “Samoan Islands” OR “Navigator Island” OR “Navigator Islands” OR “Sao Tome” OR “Saudi Arabia” OR Senegal OR Serbia OR Montenegro OR Seychelles OR “Sierra Leone” OR Singapore OR “Sri Lanka” OR Ceylon OR “Solomon Islands” OR Somalia OR “South Africa” OR Sudan OR Suriname OR Surinam OR Swaziland OR Syria OR Tajikistan OR Tadzhikistan OR Tadjikistan OR Tadzhik OR Tanzania OR Thailand OR Togo OR “Togolese Republic” OR Tokelau OR Tonga OR Trinidad OR Tobago OR Tunisia OR Turkey OR Turkmenistan OR Turkmen OR “Turks Caicos” OR “Turks and Caicos” OR Tuvalu OR Uganda OR “United Arab Emirates” OR Uruguay OR Uzbekistan OR Uzbek OR Vanuatu OR “New Hebrides” OR Venezuela OR Vietnam OR “Viet Nam” OR “Virgin Islands” OR “West Bank” OR Yemen OR Yugoslavia OR Zambia OR Zimbabwe

**DATABASES**

| Web of Science Core collection |
| --- |
| $ for wildcards  * for truncation  Both can be used with quotation marks |
| Last search date: 24 September, 2019 |
| Fields searched: topics (includes titles and abstracts)  *Indexes=SCI-EXPANDED, SSCI, A&HCI, CPCI-S, CPCI-SSH, BKCI-S, BKCI-SSH, ESCI, CCR-EXPANDED, IC Timespan=All years* |
| (TS=(standard precautions OR infection control OR infection prevention OR IPC OR asepsis OR aseptic OR sterile OR sterility OR sterilization OR sterilisation OR personal protective equipment OR PPE OR splash protection OR respiratory protection OR mask$ OR glove$ OR gown$ OR scrubs OR goggles OR eye protection OR face shield$ OR respirator$ OR Sharps OR needle$ OR syringe$ OR Water OR Sanitation OR Sanitary OR Plumbing OR Sewage OR Sewer* OR Latrine$ OR Toilet$ OR Hygiene OR Hygienic OR Shower$ OR Soap$ OR detergent$ OR Handwashing OR hand washing OR hand hygiene OR Laundry OR bedsheet$ OR bedding OR linen$ OR Waste$ OR Landfill$ OR Dump$ OR Drainage OR Wastewater OR waste water OR disposal OR lighting OR light$ OR vector control OR Vectors OR pest$ OR rodent$ OR rat$ OR insect$ OR vermin OR infest* OR fly OR flies OR mosquito OR mosquitoes OR Surface$ OR fomite$ OR Chlorine OR Disinfect* OR Cleaners OR cleaning OR cleanliness OR janitor* OR housekeep*) OR TI=(standard precautions OR infection control OR infection prevention OR IPC OR asepsis OR aseptic OR sterile OR sterility OR sterilization OR sterilisation OR personal protective equipment OR PPE OR splash protection OR respiratory protection OR mask$ OR glove$ OR gown$ OR scrubs OR goggles OR eye protection OR face shield$ OR respirator$ OR Sharps OR needle$ OR syringe$ OR Water OR Sanitation OR Sanitary OR Plumbing OR Sewage OR Sewer* OR Latrine$ OR Toilet$ OR Hygiene OR Hygienic OR Shower$ OR Soap$ OR detergent$ OR Handwashing OR hand washing OR hand hygiene OR Laundry OR bedsheet$ OR bedding OR linen$ OR Waste$ OR Landfill$ OR Dump$ OR Drainage OR Wastewater OR waste water OR disposal OR lighting OR light$ OR vector control OR Vectors OR pest$ OR rodent$ OR rat$ OR insect$ OR vermin OR infest* OR fly OR flies OR mosquito OR mosquitoes OR Surface$ OR fomite$ OR Chlorine OR Disinfect* OR Cleaners OR cleaning OR cleanliness OR janitor* OR housekeep*)) AND (TS=(healthcare OR health care OR hospital$ OR clinic$ OR health facilit* OR health center$ OR healthcenter$ OR healthcentre$ OR health centre$ OR health post$ OR healthpost$ OR health setting* OR medical facilit* OR medical center$ OR medical centre$ OR medical post$ OR medical setting* OR delivery facilit* OR delivery center$ OR delivery centre$ OR delivery clinic$ OR birth facilit* OR birth center$ OR birth centre$ OR birth clinic$ OR matern* facilit* OR matern* center$ OR matern* centre$ OR matern* clinic$ OR dental facilit* OR dental center$ OR dental centre$ OR dental clinic$ OR dispensary OR dispensaries) OR TI=(healthcare OR health care OR hospital$ OR clinic$ OR health facilit* OR health center$ OR healthcenter$ OR healthcentre$ OR health centre$ OR health post$ OR healthpost$ OR health setting* OR medical facilit* OR medical center$ OR medical centre$ OR medical post$ OR medical setting* OR delivery facilit* OR delivery center$ OR delivery centre$ OR delivery clinic$ OR birth facilit* OR birth center$ OR birth centre$ OR birth clinic$ OR matern* facilit* OR matern* center$ OR matern* centre$ OR matern* clinic$ OR dental facilit* OR dental center$ OR dental centre$ OR dental clinic$ OR dispensary OR dispensaries)) AND (TS=(Cost$ OR costing OR economic OR budget* OR Price$ OR pricing OR fee$ OR tariff$ OR loan$ OR subsidy OR subsidies OR finance$ OR financial OR financing OR fund$ OR funding OR pay OR payment$ OR investment$ OR investing OR capital OR money OR monies OR expense$ OR accounting) OR TI=(Cost$ OR costing OR economic OR budget* OR Price$ OR pricing OR fee$ OR tariff$ OR loan$ OR subsidy OR subsidies OR finance$ OR financial OR financing OR fund$ OR funding OR pay OR payment$ OR investment$ OR investing OR capital OR money OR monies OR expense$ OR accounting) OR TI==(Cost$ OR costing OR economic OR budget* OR Price$ OR pricing OR fee$ OR tariff$ OR loan$ OR subsidy OR subsidies OR finance$ OR financial OR financing OR fund$ OR funding OR pay OR payment$ OR investment$ OR investing OR capital OR money OR monies OR expense$ OR accounting) OR TI=(Cost$ OR costing OR economic OR budget* OR Price$ OR pricing OR fee$ OR tariff$ OR loan$ OR subsidy OR subsidies OR finance$ OR financial OR financing OR fund$ OR funding OR pay OR payment$ OR investment$ OR investing OR capital OR money OR monies OR expense$ OR accounting)) AND (TS=(Afghanistan OR Algeria OR Angola OR Anguilla OR Antigua OR Barbuda OR Argentina OR Armenia OR Armenian OR Aruba OR Azerbaijan OR Bahamas OR Bahrain OR Bangladesh OR Barbados OR Benin OR Byelarus OR Byelorussian OR Belarus OR Belorussian OR Belorussia OR Belize OR Bhutan OR Bolivia OR Botswana OR Brazil OR Brunei OR Burkina Faso OR Burkina Fasso OR Upper Volta OR Burundi OR Urundi OR Cambodia OR Khmer Republic OR Kampuchea OR Cameroon OR Cameroons OR Cameron OR Camerons OR Cape Verde OR Cayman Islands OR Central African Republic OR Chad OR Chile OR China OR Colombia OR Comoros OR Comoro Islands OR Comores OR Mayotte OR Congo OR Zaire OR Cook Islands OR Costa Rica OR Cote d'Ivoire OR Ivory Coast OR Croatia OR Cuba OR Cyprus OR Djibouti OR French Somaliland OR Dominica OR Dominican Republic OR East Timor OR East Timur OR Timor Leste OR Ecuador OR Egypt OR United Arab Republic OR El Salvador OR Eritrea OR Ethiopia OR Falkland Islands OR Las Malvinas OR Fiji OR Gabon OR Gabonese Republic OR Gambia OR Gaza OR Georgia Republic OR Georgian Republic OR Ghana OR Gold Coast OR Greece OR Grenada OR Guatemala OR Guinea OR Guam OR Guadeloupe OR Guiana OR Guyana OR Haiti OR Honduras OR Hong Kong OR India OR Maldives OR Indonesia OR Iran OR Iraq OR Jamaica OR Jordan OR Kazakhstan OR Kazakh OR Kenya OR Kiribati OR Korea OR Kosovo OR Kuwait OR Kyrgyzstan OR Kirghizia OR Kyrgyz Republic OR Kirghiz OR Kirgizstan OR Lao PDR OR Laos OR Lebanon OR Lesotho OR Basutoland OR Liberia OR Libya OR Macau OR Madagascar OR Malagasy Republic OR Maldives OR Malaysia OR Malaya OR Malay OR Sabah OR Sarawak OR Malawi OR Nyasaland OR Mali OR Malta OR Marshall Islands OR Martinique OR Mauritania OR Mauritius OR Agalega Islands OR Mexico OR Micronesia OR Middle East OR Mongolia OR Montserrat OR Morocco OR Ifni OR Mozambique OR Myanmar OR Myanma OR Burma OR Namibia OR Nauru OR Nepal OR Niui OR Netherlands Antilles OR New Caledonia OR Nicaragua OR Niger OR Nigeria OR Northern Mariana Islands OR Oman OR Mayotte OR Muscat OR Pakistan OR Palau OR Palestine OR Panama OR Paraguay OR Peru OR Philippines OR Philipines OR Phillipines OR Phillippines OR Polynesia OR Puerto Rico OR Qatar OR Reunion OR Rwanda OR Ruanda OR Saint Kitts OR St Kitts OR Nevis OR Saint Lucia OR St Lucia OR Saint Vincent OR St Vincent OR Grenadines OR Samoa OR Samoan Islands OR Navigator Island OR Navigator Islands OR Sao Tome OR Saudi Arabia OR Senegal OR Serbia OR Montenegro OR Seychelles OR Sierra Leone OR Singapore OR Sri Lanka OR Ceylon OR Solomon Islands OR Somalia OR South Africa OR Sudan OR Suriname OR Surinam OR Swaziland OR Syria OR Tajikistan OR Tadzhikistan OR Tadjikistan OR Tadzhik OR Tanzania OR Thailand OR Togo OR Togolese Republic OR Tokelau OR Tonga OR Trinidad OR Tobago OR Tunisia OR Turkey OR Turkmenistan OR Turkmen OR Turks Caicos OR Turks and Caicos OR Tuvalu OR Uganda OR United Arab Emirates OR Uruguay OR Uzbekistan OR Uzbek OR Vanuatu OR New Hebrides OR Venezuela OR Vietnam OR Viet Nam OR Virgin Islands OR West Bank OR Yemen OR Yugoslavia OR Zambia OR Zimbabwe) OR TI=(Afghanistan OR Algeria OR Angola OR Anguilla OR Antigua OR Barbuda OR Argentina OR Armenia OR Armenian OR Aruba OR Azerbaijan OR Bahamas OR Bahrain OR Bangladesh OR Barbados OR Benin OR Byelarus OR Byelorussian OR Belarus OR Belorussian OR Belorussia OR Belize OR Bhutan OR Bolivia OR Botswana OR Brazil OR Brunei OR Burkina Faso OR Burkina Fasso OR Upper Volta OR Burundi OR Urundi OR Cambodia OR Khmer Republic OR Kampuchea OR Cameroon OR Cameroons OR Cameron OR Camerons OR Cape Verde OR Cayman Islands OR Central African Republic OR Chad OR Chile OR China OR Colombia OR Comoros OR Comoro Islands OR Comores OR Mayotte OR Congo OR Zaire OR Cook Islands OR Costa Rica OR Cote d'Ivoire OR Ivory Coast OR Croatia OR Cuba OR Cyprus OR Djibouti OR French Somaliland OR Dominica OR Dominican Republic OR East Timor OR East Timur OR Timor Leste OR Ecuador OR Egypt OR United Arab Republic OR El Salvador OR Eritrea OR Ethiopia OR Falkland Islands OR Las Malvinas OR Fiji OR Gabon OR Gabonese Republic OR Gambia OR Gaza OR Georgia Republic OR Georgian Republic OR Ghana OR Gold Coast OR Greece OR Grenada OR Guatemala OR Guinea OR Guam OR Guadeloupe OR Guiana OR Guyana OR Haiti OR Honduras OR Hong Kong OR India OR Maldives OR Indonesia OR Iran OR Iraq OR Jamaica OR Jordan OR Kazakhstan OR Kazakh OR Kenya OR Kiribati OR Korea OR Kosovo OR Kuwait OR Kyrgyzstan OR Kirghizia OR Kyrgyz Republic OR Kirghiz OR Kirgizstan OR Lao PDR OR Laos OR Lebanon OR Lesotho OR Basutoland OR Liberia OR Libya OR Macau OR Madagascar OR Malagasy Republic OR Maldives OR Malaysia OR Malaya OR Malay OR Sabah OR Sarawak OR Malawi OR Nyasaland OR Mali OR Malta OR Marshall Islands OR Martinique OR Mauritania OR Mauritius OR Agalega Islands OR Mexico OR Micronesia OR Middle East OR Mongolia OR Montserrat OR Morocco OR Ifni OR Mozambique OR Myanmar OR Myanma OR Burma OR Namibia OR Nauru OR Nepal OR Niui OR Netherlands Antilles OR New Caledonia OR Nicaragua OR Niger OR Nigeria OR Northern Mariana Islands OR Oman OR Mayotte OR Muscat OR Pakistan OR Palau OR Palestine OR Panama OR Paraguay OR Peru OR Philippines OR Philipines OR Phillipines OR Phillippines OR Polynesia OR Puerto Rico OR Qatar OR Reunion OR Rwanda OR Ruanda OR Saint Kitts OR St Kitts OR Nevis OR Saint Lucia OR St Lucia OR Saint Vincent OR St Vincent OR Grenadines OR Samoa OR Samoan Islands OR Navigator Island OR Navigator Islands OR Sao Tome OR Saudi Arabia OR Senegal OR Serbia OR Montenegro OR Seychelles OR Sierra Leone OR Singapore OR Sri Lanka OR Ceylon OR Solomon Islands OR Somalia OR South Africa OR Sudan OR Suriname OR Surinam OR Swaziland OR Syria OR Tajikistan OR Tadzhikistan OR Tadjikistan OR Tadzhik OR Tanzania OR Thailand OR Togo OR Togolese Republic OR Tokelau OR Tonga OR Trinidad OR Tobago OR Tunisia OR Turkey OR Turkmenistan OR Turkmen OR Turks Caicos OR Turks and Caicos OR Tuvalu OR Uganda OR United Arab Emirates OR Uruguay OR Uzbekistan OR Uzbek OR Vanuatu OR New Hebrides OR Venezuela OR Vietnam OR Viet Nam OR Virgin Islands OR West Bank OR Yemen OR Yugoslavia OR Zambia OR Zimbabwe)) |

| PubMed |
| --- |
| * truncation only, no single character truncation  Cannot be used in quotation marks. Truncation can be used at the end of a phrase to keep terms together (e.g. cost analy* to pick up “cost analysis” “cost analytics”) but not in the middle of a phrase (e.g. cost* analysis will pick up ((cost OR costs OR cost-effective…….) AND (analysis)) anywhere, not as a combined phrase) |
| Last search date: 24 September, 2019 |
| Fields searched: Title/Abstract |
| ((((“standard precautions”[Title/Abstract] OR “infection control”[Title/Abstract] OR “infection prevention”[Title/Abstract] OR IPC[Title/Abstract] OR asepsis[Title/Abstract] OR aseptic[Title/Abstract] OR sterile[Title/Abstract] OR sterility[Title/Abstract] OR sterilization[Title/Abstract] OR sterilisation[Title/Abstract] OR “personal protective equipment”[Title/Abstract] OR PPE[Title/Abstract] OR “splash protection”[Title/Abstract] OR “respiratory protection”[Title/Abstract] OR mask[Title/Abstract] OR masks[Title/Abstract] OR glove[Title/Abstract] OR gloves[Title/Abstract] OR gown[Title/Abstract] OR gowns[Title/Abstract] OR scrubs[Title/Abstract] OR goggles[Title/Abstract] OR “eye protection”[Title/Abstract] OR “face shield”[Title/Abstract] OR “face shields”[Title/Abstract] OR respirator[Title/Abstract] OR respirators[Title/Abstract] OR Sharps[Title/Abstract] OR needle[Title/Abstract] OR needles[Title/Abstract] OR syringe[Title/Abstract] OR syringes[Title/Abstract] OR Water[Title/Abstract] OR Sanitation[Title/Abstract] OR Sanitary[Title/Abstract] OR Plumbing[Title/Abstract] OR Sewage[Title/Abstract] OR Sewer*[Title/Abstract] OR Latrine[Title/Abstract] OR latrines[Title/Abstract] OR Toilet[Title/Abstract] OR toilets[Title/Abstract] OR Hygiene[Title/Abstract] OR Hygienic[Title/Abstract] OR Shower[Title/Abstract] OR showers[Title/Abstract] OR Soap[Title/Abstract] OR soaps[Title/Abstract] OR detergent[Title/Abstract] OR detergents[Title/Abstract] OR Handwashing[Title/Abstract] OR “hand washing”[Title/Abstract] OR “hand hygiene”[Title/Abstract] OR Laundry[Title/Abstract] OR bedsheet[Title/Abstract] OR bedsheets[Title/Abstract] OR bedding[Title/Abstract] OR linen[Title/Abstract] OR linens[Title/Abstract] OR Waste[Title/Abstract] OR wastes[Title/Abstract] OR Landfill[Title/Abstract] OR landfills[Title/Abstract] OR Dump[Title/Abstract] OR dumps[Title/Abstract] OR Drainage[Title/Abstract] OR Wastewater[Title/Abstract] OR “waste water”[Title/Abstract] OR disposal[Title/Abstract] OR lighting[Title/Abstract] OR light[Title/Abstract] OR lights[Title/Abstract] OR “vector control”[Title/Abstract] OR Vectors[Title/Abstract] OR pest[Title/Abstract] OR pests[Title/Abstract] OR rodent[Title/Abstract] OR rodents[Title/Abstract] OR rat[Title/Abstract] OR rats[Title/Abstract] OR insect[Title/Abstract] OR insects[Title/Abstract] OR vermin[Title/Abstract] OR infest*[Title/Abstract] OR fly[Title/Abstract] OR flies[Title/Abstract] OR mosquito[Title/Abstract] OR mosquitoes[Title/Abstract] OR Surface[Title/Abstract] OR surfaces[Title/Abstract] OR fomite$[Title/Abstract] OR Chlorine[Title/Abstract] OR Disinfect*[Title/Abstract] OR cleaner[Title/Abstract] OR Cleaners[Title/Abstract] OR cleaning[Title/Abstract] OR cleanliness[Title/Abstract] OR janitor*[Title/Abstract] OR housekeep*[Title/Abstract])) AND (healthcare[Title/Abstract] OR “health care”[Title/Abstract] OR hospital[Title/Abstract] OR hospitals[Title/Abstract] OR clinic[Title/Abstract] OR clinics[Title/Abstract] OR “health facility”[Title/Abstract] OR “health facilities”[Title/Abstract] OR “health center”[Title/Abstract] OR “health centers”[Title/Abstract] OR healthcenter[Title/Abstract] OR healthcenters[Title/Abstract] OR healthcentre[Title/Abstract] OR healthcentre[Title/Abstract] OR “health centre”[Title/Abstract] OR “health centres”[Title/Abstract] OR “health post”[Title/Abstract] OR “health post”[Title/Abstract] OR healthpost[Title/Abstract] OR healthposts[Title/Abstract] OR “health setting”[Title/Abstract] OR “health settings”[Title/Abstract] OR “medical facility”[Title/Abstract] OR “medical facilities”[Title/Abstract] OR “medical center”[Title/Abstract] OR “medical centers”[Title/Abstract] OR “medical centre”[Title/Abstract] OR “medical centres”[Title/Abstract] OR “medical post”[Title/Abstract] OR “medical posts”[Title/Abstract] OR “medical setting”[Title/Abstract] OR “medical settings”[Title/Abstract] OR “delivery facility”[Title/Abstract] OR “delivery facilities”[Title/Abstract] OR “delivery center”[Title/Abstract] OR “delivery centers”[Title/Abstract] OR “delivery centre”[Title/Abstract] OR “delivery centres”[Title/Abstract] OR “delivery clinic”[Title/Abstract] OR “delivery clinics”[Title/Abstract] OR “birth facility”[Title/Abstract] OR “birth facilities”[Title/Abstract] OR “birth center”[Title/Abstract] OR “birth centers”[Title/Abstract] OR “birth centre”[Title/Abstract] OR “birth centres”[Title/Abstract] OR “birth clinic”[Title/Abstract] OR “birth clincs”[Title/Abstract] OR “maternal facility”[Title/Abstract] OR “maternity facility”[Title/Abstract] OR “maternal facilities”[Title/Abstract] OR “maternity facilities”[Title/Abstract] OR “maternal center”[Title/Abstract] OR “maternity center”[Title/Abstract] OR “maternal centers”[Title/Abstract] OR “maternity centers”[Title/Abstract] OR “maternal centre”[Title/Abstract] OR “maternal centers”[Title/Abstract] OR “maternity clinic”[Title/Abstract] OR “maternity clinics”[Title/Abstract] OR “maternal clinic”[Title/Abstract] OR “maternal clinics”[Title/Abstract] OR “maternity home”[Title/Abstract] OR “maternity homes”[Title/Abstract] OR “maternity waiting homes”[Title/Abstract] OR “dental facility”[Title/Abstract] OR “dental facilities”[Title/Abstract] OR “dental center”[Title/Abstract] OR “dental centers”[Title/Abstract] OR “dental centre”[Title/Abstract] OR “dental centres”[Title/Abstract] OR “dental clinic”[Title/Abstract] OR “dental clinics”[Title/Abstract] OR “dispensary”[Title/Abstract] OR “dispensaries”[Title/Abstract])) AND (Cost[Title/Abstract] OR costs[Title/Abstract] OR costing[Title/Abstract] OR economic[Title/Abstract] OR budget[Title/Abstract] OR budgets[Title/Abstract] OR budgetary[Title/Abstract] OR budgeting[Title/Abstract] OR Price[Title/Abstract] OR prices[Title/Abstract] OR pricing[Title/Abstract] OR fee[Title/Abstract] OR fees[Title/Abstract] OR tariff[Title/Abstract] OR tariffs[Title/Abstract] OR loan[Title/Abstract] OR loans[Title/Abstract] OR subsidy[Title/Abstract] OR subsidies[Title/Abstract] OR finance[Title/Abstract] OR finances[Title/Abstract] OR financial[Title/Abstract] OR financing[Title/Abstract] OR fund[Title/Abstract] OR funds[Title/Abstract] OR funding[Title/Abstract] OR pay[Title/Abstract] OR payment[Title/Abstract] OR payments[Title/Abstract] OR investment[Title/Abstract] OR investments[Title/Abstract] OR investing[Title/Abstract] OR capital[Title/Abstract] OR money[Title/Abstract] OR monies[Title/Abstract] OR expense[Title/Abstract] OR expenses[Title/Abstract] OR accounting[Title/Abstract])) AND (Afghanistan[Title/Abstract] OR Algeria[Title/Abstract] OR Angola[Title/Abstract] OR Anguilla[Title/Abstract] OR Antigua[Title/Abstract] OR Barbuda[Title/Abstract] OR Argentina[Title/Abstract] OR Armenia[Title/Abstract] OR Armenian[Title/Abstract] OR Aruba[Title/Abstract] OR Azerbaijan[Title/Abstract] OR Bahamas[Title/Abstract] OR Bahrain[Title/Abstract] OR Bangladesh[Title/Abstract] OR Barbados[Title/Abstract] OR Benin[Title/Abstract] OR Byelarus[Title/Abstract] OR Byelorussian[Title/Abstract] OR Belarus[Title/Abstract] OR Belorussian[Title/Abstract] OR Belorussia[Title/Abstract] OR Belize[Title/Abstract] OR Bhutan[Title/Abstract] OR Bolivia[Title/Abstract] OR Botswana[Title/Abstract] OR Brazil[Title/Abstract] OR Brunei[Title/Abstract] OR “Burkina Faso”[Title/Abstract] OR “Burkina Fasso”[Title/Abstract] OR “Upper Volta”[Title/Abstract] OR Burundi[Title/Abstract] OR Urundi[Title/Abstract] OR Cambodia[Title/Abstract] OR “Khmer Republic”[Title/Abstract] OR Kampuchea[Title/Abstract] OR Cameroon[Title/Abstract] OR Cameroons[Title/Abstract] OR Cameron[Title/Abstract] OR Camerons[Title/Abstract] OR “Cape Verde”[Title/Abstract] OR “Cayman Islands”[Title/Abstract] OR “Central African Republic”[Title/Abstract] OR Chad[Title/Abstract] OR Chile[Title/Abstract] OR China[Title/Abstract] OR Colombia[Title/Abstract] OR Comoros[Title/Abstract] OR “Comoro Islands”[Title/Abstract] OR Comores[Title/Abstract] OR Mayotte[Title/Abstract] OR Congo[Title/Abstract] OR Zaire[Title/Abstract] OR “Cook Islands”[Title/Abstract] OR “Costa Rica”[Title/Abstract] OR “Cote d'Ivoire”[Title/Abstract] OR “Ivory Coast”[Title/Abstract] OR Croatia[Title/Abstract] OR Cuba[Title/Abstract] OR Cyprus[Title/Abstract] OR Djibouti[Title/Abstract] OR “French Somaliland”[Title/Abstract] OR Dominica[Title/Abstract] OR “Dominican Republic”[Title/Abstract] OR “East Timor”[Title/Abstract] OR “East Timur”[Title/Abstract] OR “Timor Leste”[Title/Abstract] OR Ecuador[Title/Abstract] OR Egypt[Title/Abstract] OR “United Arab Republic”[Title/Abstract] OR “El Salvador”[Title/Abstract] OR Eritrea[Title/Abstract] OR Ethiopia[Title/Abstract] OR “Falkland Islands”[Title/Abstract] OR “Las Malvinas”[Title/Abstract] OR Fiji[Title/Abstract] OR Gabon[Title/Abstract] OR “Gabonese Republic”[Title/Abstract] OR Gambia[Title/Abstract] OR Gaza[Title/Abstract] OR “Georgia Republic”[Title/Abstract] OR “Georgian Republic”[Title/Abstract] OR Ghana[Title/Abstract] OR “Gold Coast”[Title/Abstract] OR Greece[Title/Abstract] OR Grenada[Title/Abstract] OR Guatemala[Title/Abstract] OR Guinea[Title/Abstract] OR Guam[Title/Abstract] OR Guadeloupe[Title/Abstract] OR Guiana[Title/Abstract] OR Guyana[Title/Abstract] OR Haiti[Title/Abstract] OR Honduras[Title/Abstract] OR “Hong Kong”[Title/Abstract] OR India[Title/Abstract] OR Maldives[Title/Abstract] OR Indonesia[Title/Abstract] OR Iran[Title/Abstract] OR Iraq[Title/Abstract] OR Jamaica[Title/Abstract] OR Jordan[Title/Abstract] OR Kazakhstan[Title/Abstract] OR Kazakh[Title/Abstract] OR Kenya[Title/Abstract] OR Kiribati[Title/Abstract] OR Korea[Title/Abstract] OR Kosovo[Title/Abstract] OR Kuwait[Title/Abstract] OR Kyrgyzstan[Title/Abstract] OR Kirghizia[Title/Abstract] OR “Kyrgyz Republic”[Title/Abstract] OR Kirghiz[Title/Abstract] OR Kirgizstan[Title/Abstract] OR “Lao PDR”[Title/Abstract] OR Laos[Title/Abstract] OR Lebanon[Title/Abstract] OR Lesotho[Title/Abstract] OR Basutoland[Title/Abstract] OR Liberia[Title/Abstract] OR Libya[Title/Abstract] OR Macau[Title/Abstract] OR Madagascar[Title/Abstract] OR “Malagasy Republic”[Title/Abstract] OR Maldives[Title/Abstract] OR Malaysia[Title/Abstract] OR Malaya[Title/Abstract] OR Malay[Title/Abstract] OR Sabah[Title/Abstract] OR Sarawak[Title/Abstract] OR Malawi[Title/Abstract] OR Nyasaland[Title/Abstract] OR Mali[Title/Abstract] OR Malta[Title/Abstract] OR “Marshall Islands”[Title/Abstract] OR Martinique[Title/Abstract] OR Mauritania[Title/Abstract] OR Mauritius[Title/Abstract] OR “Agalega Islands”[Title/Abstract] OR Mexico[Title/Abstract] OR Micronesia[Title/Abstract] OR “Middle East”[Title/Abstract] OR Mongolia[Title/Abstract] OR Montserrat[Title/Abstract] OR Morocco[Title/Abstract] OR Ifni[Title/Abstract] OR Mozambique[Title/Abstract] OR Myanmar[Title/Abstract] OR Myanma[Title/Abstract] OR Burma[Title/Abstract] OR Namibia[Title/Abstract] OR Nauru[Title/Abstract] OR Nepal[Title/Abstract] OR Niui[Title/Abstract] OR “Netherlands Antilles”[Title/Abstract] OR “New Caledonia”[Title/Abstract] OR Nicaragua[Title/Abstract] OR Niger[Title/Abstract] OR Nigeria[Title/Abstract] OR “Northern Mariana Islands”[Title/Abstract] OR Oman[Title/Abstract] OR Mayotte[Title/Abstract] OR Muscat[Title/Abstract] OR Pakistan[Title/Abstract] OR Palau[Title/Abstract] OR Palestine[Title/Abstract] OR Panama[Title/Abstract] OR Paraguay[Title/Abstract] OR Peru[Title/Abstract] OR Philippines[Title/Abstract] OR Philipines[Title/Abstract] OR Phillipines[Title/Abstract] OR Phillippines[Title/Abstract] OR Polynesia[Title/Abstract] OR “Puerto Rico”[Title/Abstract] OR Qatar[Title/Abstract] OR Reunion[Title/Abstract] OR Rwanda[Title/Abstract] OR Ruanda[Title/Abstract] OR “Saint Kitts”[Title/Abstract] OR “St Kitts”[Title/Abstract] OR Nevis[Title/Abstract] OR “Saint Lucia”[Title/Abstract] OR “St Lucia”[Title/Abstract] OR “Saint Vincent”[Title/Abstract] OR “St Vincent”[Title/Abstract] OR Grenadines[Title/Abstract] OR Samoa[Title/Abstract] OR “Samoan Islands”[Title/Abstract] OR “Navigator Island”[Title/Abstract] OR “Navigator Islands”[Title/Abstract] OR “Sao Tome”[Title/Abstract] OR “Saudi Arabia”[Title/Abstract] OR Senegal[Title/Abstract] OR Serbia[Title/Abstract] OR Montenegro[Title/Abstract] OR Seychelles[Title/Abstract] OR “Sierra Leone”[Title/Abstract] OR Singapore[Title/Abstract] OR “Sri Lanka”[Title/Abstract] OR Ceylon[Title/Abstract] OR “Solomon Islands”[Title/Abstract] OR Somalia[Title/Abstract] OR “South Africa”[Title/Abstract] OR Sudan[Title/Abstract] OR Suriname[Title/Abstract] OR Surinam[Title/Abstract] OR Swaziland[Title/Abstract] OR Syria[Title/Abstract] OR Tajikistan[Title/Abstract] OR Tadzhikistan[Title/Abstract] OR Tadjikistan[Title/Abstract] OR Tadzhik[Title/Abstract] OR Tanzania[Title/Abstract] OR Thailand[Title/Abstract] OR Togo[Title/Abstract] OR “Togolese Republic”[Title/Abstract] OR Tokelau[Title/Abstract] OR Tonga[Title/Abstract] OR Trinidad[Title/Abstract] OR Tobago[Title/Abstract] OR Tunisia[Title/Abstract] OR Turkey[Title/Abstract] OR Turkmenistan[Title/Abstract] OR Turkmen[Title/Abstract] OR “Turks Caicos”[Title/Abstract] OR “Turks[Title/Abstract] AND Caicos”[Title/Abstract] OR Tuvalu[Title/Abstract] OR Uganda[Title/Abstract] OR “United Arab Emirates”[Title/Abstract] OR Uruguay[Title/Abstract] OR Uzbekistan[Title/Abstract] OR Uzbek[Title/Abstract] OR Vanuatu[Title/Abstract] OR “New Hebrides”[Title/Abstract] OR Venezuela[Title/Abstract] OR Vietnam[Title/Abstract] OR “Viet Nam”[Title/Abstract] OR “Virgin Islands”[Title/Abstract] OR “West Bank”[Title/Abstract] OR Yemen[Title/Abstract] OR Yugoslavia[Title/Abstract] OR Zambia[Title/Abstract] OR Zimbabwe[Title/Abstract]) |

| Scopus |
| --- |
| $ for wildcards (can be any letter or nothing)  * for truncation  Both can be used in quotation marks |
| Last search date: 24 September, 2019 |
| Fields searched: titles, abstracts, and keywords |
| TITLE-ABS-KEY ( "standard precautions"  OR  "infection control"  OR  "infection prevention"  OR  ipc  OR  asepsis  OR  aseptic  OR  sterile  OR  sterility  OR  sterilization  OR  sterilisation  OR  "personal protective equipment"  OR  ppe  OR  "splash protection"  OR  "respiratory protection"  OR  mask$  OR  glove$  OR  gown$  OR  scrubs  OR  goggles  OR  "eye protection"  OR  "face shield$"  OR  respirator$  OR  sharps  OR  needle$  OR  syringe$  OR  water  OR  sanitation  OR  sanitary  OR  plumbing  OR  sewage  OR  sewer*  OR  latrine$  OR  toilet$  OR  hygiene  OR  hygienic  OR  shower$  OR  soap$  OR  detergent$  OR  handwashing  OR  "hand washing"  OR  "hand hygiene"  OR  laundry  OR  bedsheet$  OR  bedding  OR  linen$  OR  waste$  OR  landfill$  OR  dump$  OR  drainage  OR  wastewater  OR  "waste water"  OR  disposal  OR  lighting  OR  light$  OR  "vector control" OR  vectors  OR  pest$  OR  rodent$  OR  rat$  OR  insect$  OR  vermin  OR  infest*  OR  fly  OR  flies  OR  mosquito  OR  mosquitoes  OR  surface$  OR  fomite$  OR  chlorine  OR  disinfect*  OR  cleaners  OR  cleaning  OR  cleanliness  OR  janitor*  OR  housekeep* )  AND  TITLE-ABS-KEY ( healthcare  OR  "health care"  OR  hospital$  OR  clinic$  OR  "health facilit*"  OR  "health center$"  OR  healthcenter$  OR  healthcentre$  OR  "health centre$"  OR  "health post$"  OR  healthpost$  OR  "health setting*"  OR  "medical facilit*"  OR  "medical center$"  OR  "medical centre$"  OR  "medical post$"  OR  "medical setting*"  OR  "delivery facilit*"  OR  "delivery center$"  OR  "delivery centre$"  OR  "delivery clinic$"  OR  "birth facilit*"  OR  "birth center$"  OR  "birth centre$"  OR  "birth clinic$"  OR  "matern* facilit*"  OR  "matern* center$"  OR  "matern* centre$"  OR  "matern* clinic$"  OR  "dental facilit*"  OR  "dental center$"  OR  "dental centre$"  OR  "dental clinic$"  OR  dispensary  OR  dispensaries )  AND  TITLE-ABS-KEY ( cost$  OR  costing  OR  economic  OR  budget*  OR  price$  OR  pricing  OR  fee$  OR  tariff$  OR  loan$  OR  subsidy  OR  subsidies  OR  finance$  OR  financial  OR  financing  OR  fund$  OR  funding  OR  pay  OR  payment$  OR  investment$  OR  investing  OR  capital  OR  money  OR  monies  OR  expense$ OR accounting )  AND  TITLE-ABS-KEY ( afghanistan  OR  algeria  OR  angola  OR  anguilla  OR  antigua  OR  barbuda  OR  argentina  OR  armenia  OR  armenian  OR  aruba  OR  azerbaijan  OR  bahamas  OR  bahrain  OR  bangladesh  OR  barbados  OR  benin  OR  byelarus  OR  byelorussian  OR  belarus  OR  belorussian  OR  belorussia  OR  belize  OR  bhutan  OR  bolivia  OR  botswana  OR  brazil  OR  brunei  OR  "Burkina Faso"  OR  "Burkina Fasso"  OR  "Upper Volta"  OR  burundi  OR  urundi  OR  cambodia  OR  "Khmer Republic"  OR  kampuchea  OR  cameroon  OR  cameroons  OR  cameron  OR  camerons  OR  "Cape Verde"  OR  "Cayman Islands"  OR  "Central African Republic"  OR  chad  OR  chile  OR  china  OR  colombia  OR  comoros  OR  "Comoro Islands"  OR  comores  OR  mayotte  OR  congo  OR  zaire  OR  "Cook Islands"  OR  "Costa Rica"  OR  "Cote d'Ivoire"  OR  "Ivory Coast"  OR  croatia  OR  cuba  OR  cyprus  OR  djibouti  OR  "French Somaliland"  OR  dominica  OR  "Dominican Republic"  OR  "East Timor"  OR  "East Timur"  OR  "Timor Leste"  OR  ecuador  OR  egypt  OR  "United Arab Republic"  OR  "El Salvador"  OR  eritrea  OR  ethiopia  OR  "Falkland Islands"  OR  "Las Malvinas"  OR  fiji  OR  gabon  OR  "Gabonese Republic"  OR  gambia  OR  gaza  OR  "Georgia Republic"  OR  "Georgian Republic"  OR  ghana  OR  "Gold Coast"  OR  greece  OR  grenada  OR  guatemala  OR  guinea  OR  guam  OR  guadeloupe  OR  guiana  OR  guyana  OR  haiti  OR  honduras  OR  "Hong Kong"  OR  india  OR  maldives  OR  indonesia  OR  iran  OR  iraq  OR  jamaica  OR  jordan  OR  kazakhstan  OR  kazakh  OR  kenya  OR  kiribati  OR  korea  OR  kosovo  OR  kuwait  OR  kyrgyzstan  OR  kirghizia  OR  "Kyrgyz Republic"  OR  kirghiz  OR  kirgizstan  OR  "Lao PDR"  OR  laos  OR  lebanon  OR  lesotho  OR  basutoland  OR  liberia  OR  libya  OR  macau  OR  madagascar  OR  "Malagasy Republic"  OR  maldives  OR  malaysia  OR  malaya  OR  malay  OR  sabah  OR  sarawak  OR  malawi  OR  nyasaland  OR  mali  OR  malta  OR  "Marshall Islands"  OR  martinique  OR  mauritania  OR  mauritius  OR  "Agalega Islands"  OR  mexico  OR  micronesia  OR  "Middle East"  OR  mongolia  OR  montserrat  OR  morocco  OR  ifni  OR  mozambique  OR  myanmar  OR  myanma  OR  burma  OR  namibia  OR  nauru  OR  nepal  OR  niui  OR  "Netherlands Antilles"  OR  "New Caledonia"  OR  nicaragua  OR  niger  OR  nigeria  OR  "Northern Mariana Islands"  OR  oman  OR  mayotte  OR  muscat  OR  pakistan  OR  palau  OR  palestine  OR  panama  OR  paraguay  OR  peru  OR  philippines  OR  philipines  OR  phillipines  OR  phillippines  OR  polynesia  OR  "Puerto Rico"  OR  qatar  OR  reunion  OR  rwanda  OR  ruanda  OR  "Saint Kitts"  OR  "St Kitts"  OR  nevis  OR  "Saint Lucia"  OR  "St Lucia"  OR  "Saint Vincent"  OR  "St Vincent"  OR  grenadines  OR  samoa  OR  "Samoan Islands"  OR  "Navigator Island"  OR  "Navigator Islands"  OR  "Sao Tome"  OR  "Saudi Arabia"  OR  senegal  OR  serbia  OR  montenegro  OR  seychelles  OR  "Sierra Leone"  OR  singapore  OR  "Sri Lanka"  OR  ceylon  OR  "Solomon Islands"  OR  somalia  OR  "South Africa"  OR  sudan  OR  suriname  OR  surinam  OR  swaziland  OR  syria  OR  tajikistan  OR  tadzhikistan  OR  tadjikistan  OR  tadzhik  OR  tanzania  OR  thailand  OR  togo  OR  "Togolese Republic"  OR  tokelau  OR  tonga  OR  trinidad  OR  tobago  OR  tunisia  OR  turkey  OR  turkmenistan  OR  turkmen  OR  "Turks Caicos"  OR  "Turks and Caicos"  OR  tuvalu  OR  uganda  OR  "United Arab Emirates"  OR  uruguay  OR  uzbekistan  OR  uzbek  OR  vanuatu  OR  "New Hebrides"  OR  venezuela  OR  vietnam  OR  "Viet Nam"  OR  "Virgin Islands"  OR  "West Bank"  OR  yemen  OR  yugoslavia  OR  zambia  OR  zimbabwe) |

| Ebsco Business Source Premier and Global Health |
| --- |
| Automatically searches for plurals BUT not in quotation marked phrases  Can use # to replace single character or leave blank, but voids lemmatization function  * used for truncation, works in quotes  Both work in quotes |
| Last search date: 24 September, 2019 |
| Fields searched: title, abstract, key words |
| **( KW (**“standard precautions” OR “infection control” OR “infection prevention” OR IPC OR asepsis OR aseptic OR sterile OR sterility OR sterilization OR sterilisation OR “personal protective equipment” OR PPE OR “splash protection” OR “respiratory protection” OR mask# OR glove# OR gown# OR scrubs OR goggles OR “eye protection” OR “face shield#” OR respirator# OR Sharps OR needle# OR syringe# OR Water OR Sanitation OR Sanitary OR Plumbing OR Sewage OR Sewer* OR Latrine# OR Toilet# OR Hygiene OR Hygienic OR Shower# OR Soap# OR detergent# OR Handwashing OR “hand washing” OR “hand hygiene” OR Laundry OR bedsheet# OR bedding OR linen# OR Waste# OR Landfill# OR Dump# OR Drainage OR Wastewater OR “waste water” OR disposal OR lighting OR light# OR “vector control” OR Vectors OR pest# OR rodent# OR rat# OR insect# OR vermin OR infest* OR fly OR flies OR mosquito OR mosquitoes OR Surface# OR fomite# OR Chlorine OR Disinfect* OR Cleaners OR cleaning OR cleanliness OR janitor* OR housekeep***) OR AB (**“standard precautions” OR “infection control” OR “infection prevention” OR IPC OR asepsis OR aseptic OR sterile OR sterility OR sterilization OR sterilisation OR “personal protective equipment” OR PPE OR “splash protection” OR “respiratory protection” OR mask# OR glove# OR gown# OR scrubs OR goggles OR “eye protection” OR “face shield#” OR respirator# OR Sharps OR needle# OR syringe# OR Water OR Sanitation OR Sanitary OR Plumbing OR Sewage OR Sewer* OR Latrine# OR Toilet# OR Hygiene OR Hygienic OR Shower# OR Soap# OR detergent# OR Handwashing OR “hand washing” OR “hand hygiene” OR Laundry OR bedsheet# OR bedding OR linen# OR Waste# OR Landfill# OR Dump# OR Drainage OR Wastewater OR “waste water” OR disposal OR lighting OR light# OR “vector control” OR Vectors OR pest# OR rodent# OR rat# OR insect# OR vermin OR infest* OR fly OR flies OR mosquito OR mosquitoes OR Surface# OR fomite# OR Chlorine OR Disinfect* OR Cleaners OR cleaning OR cleanliness OR janitor* OR housekeep***) OR TI (**“standard precautions” OR “infection control” OR “infection prevention” OR IPC OR asepsis OR aseptic OR sterile OR sterility OR sterilization OR sterilisation OR “personal protective equipment” OR PPE OR “splash protection” OR “respiratory protection” OR mask# OR glove# OR gown# OR scrubs OR goggles OR “eye protection” OR “face shield#” OR respirator# OR Sharps OR needle# OR syringe# OR Water OR Sanitation OR Sanitary OR Plumbing OR Sewage OR Sewer* OR Latrine# OR Toilet# OR Hygiene OR Hygienic OR Shower# OR Soap# OR detergent# OR Handwashing OR “hand washing” OR “hand hygiene” OR Laundry OR bedsheet# OR bedding OR linen# OR Waste# OR Landfill# OR Dump# OR Drainage OR Wastewater OR “waste water” OR disposal OR lighting OR light# OR “vector control” OR Vectors OR pest# OR rodent# OR rat# OR insect# OR vermin OR infest* OR fly OR flies OR mosquito OR mosquitoes OR Surface# OR fomite# OR Chlorine OR Disinfect* OR Cleaners OR cleaning OR cleanliness OR janitor* OR housekeep***) ) AND ( KW (**healthcare OR “health care” OR hospital# OR clinic# OR “health facilit*” OR “health center#” OR healthcenter# OR healthcentre# OR “health centre#” OR “health post#” OR healthpost# OR “health setting*” OR “medical facilit*” OR “medical center#” OR “medical centre#” OR “medical post#” OR “medical setting*” OR “delivery facilit*” OR “delivery center#” OR “delivery centre#” OR “delivery clinic#” OR “birth facilit*” OR “birth center#” OR “birth centre#” OR “birth clinic#” OR “matern* facilit*” OR “matern* center#” OR “matern* centre#” OR “matern* clinic#” OR “dental facilit*” OR “dental center#” OR “dental centre#” OR “dental clinic#” OR dispensary OR dispensaries**) OR AB (**healthcare OR “health care” OR hospital# OR clinic# OR “health facilit*” OR “health center#” OR healthcenter# OR healthcentre# OR “health centre#” OR “health post#” OR healthpost# OR “health setting*” OR “medical facilit*” OR “medical center#” OR “medical centre#” OR “medical post#” OR “medical setting*” OR “delivery facilit*” OR “delivery center#” OR “delivery centre#” OR “delivery clinic#” OR “birth facilit*” OR “birth center#” OR “birth centre#” OR “birth clinic#” OR “matern* facilit*” OR “matern* center#” OR “matern* centre#” OR “matern* clinic#” OR “dental facilit*” OR “dental center#” OR “dental centre#” OR “dental clinic#” OR dispensary OR dispensaries**) OR TI (**healthcare OR “health care” OR hospital# OR clinic# OR “health facilit*” OR “health center#” OR healthcenter# OR healthcentre# OR “health centre#” OR “health post#” OR healthpost# OR “health setting*” OR “medical facilit*” OR “medical center#” OR “medical centre#” OR “medical post#” OR “medical setting*” OR “delivery facilit*” OR “delivery center#” OR “delivery centre#” OR “delivery clinic#” OR “birth facilit*” OR “birth center#” OR “birth centre#” OR “birth clinic#” OR “matern* facilit*” OR “matern* center#” OR “matern* centre#” OR “matern* clinic#” OR “dental facilit*” OR “dental center#” OR “dental centre#” OR “dental clinic#” OR dispensary OR dispensaries**) ) AND ( KW (**Cost# OR costing OR economic OR budget* OR Price# OR pricing OR fee# OR tariff# OR loan# OR subsidy OR subsidies OR finance# OR financial OR financing OR fund# OR funding OR pay OR payment# OR investment# OR investing OR capital OR money OR monies OR expense# OR accounting  **) OR AB (**Cost# OR costing OR economic OR budget* OR Price# OR pricing OR fee# OR tariff# OR loan# OR subsidy OR subsidies OR finance# OR financial OR financing OR fund# OR funding OR pay OR payment# OR investment# OR investing OR capital OR money OR monies OR expense#**) OR TI (**Cost# OR costing OR economic OR budget* OR Price# OR pricing OR fee# OR tariff# OR loan# OR subsidy OR subsidies OR finance# OR financial OR financing OR fund# OR funding OR pay OR payment# OR investment# OR investing OR capital OR money OR monies OR expense# OR accounting**) ) AND ( KW (**Afghanistan OR Algeria OR Angola OR Anguilla OR Antigua OR Barbuda OR Argentina OR Armenia OR Armenian OR Aruba OR Azerbaijan OR Bahamas OR Bahrain OR Bangladesh OR Barbados OR Benin OR Byelarus OR Byelorussian OR Belarus OR Belorussian OR Belorussia OR Belize OR Bhutan OR Bolivia OR Botswana OR Brazil OR Brunei OR “Burkina Faso” OR “Burkina Fasso” OR “Upper Volta” OR Burundi OR Urundi OR Cambodia OR “Khmer Republic” OR Kampuchea OR Cameroon OR Cameroons OR Cameron OR Camerons OR “Cape Verde” OR “Cayman Islands” OR “Central African Republic” OR Chad OR Chile OR China OR Colombia OR Comoros OR “Comoro Islands” OR Comores OR Mayotte OR Congo OR Zaire OR “Cook Islands” OR “Costa Rica” OR “Cote d'Ivoire” OR “Ivory Coast” OR Croatia OR Cuba OR Cyprus OR Djibouti OR “French Somaliland” OR Dominica OR “Dominican Republic” OR “East Timor” OR “East Timur” OR “Timor Leste” OR Ecuador OR Egypt OR “United Arab Republic” OR “El Salvador” OR Eritrea OR Ethiopia OR “Falkland Islands” OR “Las Malvinas” OR Fiji OR Gabon OR “Gabonese Republic” OR Gambia OR Gaza OR “Georgia Republic” OR “Georgian Republic” OR Ghana OR “Gold Coast” OR Greece OR Grenada OR Guatemala OR Guinea OR Guam OR Guadeloupe OR Guiana OR Guyana OR Haiti OR Honduras OR “Hong Kong” OR India OR Maldives OR Indonesia OR Iran OR Iraq OR Jamaica OR Jordan OR Kazakhstan OR Kazakh OR Kenya OR Kiribati OR Korea OR Kosovo OR Kuwait OR Kyrgyzstan OR Kirghizia OR “Kyrgyz Republic” OR Kirghiz OR Kirgizstan OR “Lao PDR” OR Laos OR Lebanon OR Lesotho OR Basutoland OR Liberia OR Libya OR Macau OR Madagascar OR “Malagasy Republic” OR Maldives OR Malaysia OR Malaya OR Malay OR Sabah OR Sarawak OR Malawi OR Nyasaland OR Mali OR Malta OR “Marshall Islands” OR Martinique OR Mauritania OR Mauritius OR “Agalega Islands” OR Mexico OR Micronesia OR “Middle East” OR Mongolia OR Montserrat OR Morocco OR Ifni OR Mozambique OR Myanmar OR Myanma OR Burma OR Namibia OR Nauru OR Nepal OR Niui OR “Netherlands Antilles” OR “New Caledonia” OR Nicaragua OR Niger OR Nigeria OR “Northern Mariana Islands” OR Oman OR Mayotte OR Muscat OR Pakistan OR Palau OR Palestine OR Panama OR Paraguay OR Peru OR Philippines OR Philipines OR Phillipines OR Phillippines OR Polynesia OR “Puerto Rico” OR Qatar OR Reunion OR Rwanda OR Ruanda OR “Saint Kitts” OR “St Kitts” OR Nevis OR “Saint Lucia” OR “St Lucia” OR “Saint Vincent” OR “St Vincent” OR Grenadines OR Samoa OR “Samoan Islands” OR “Navigator Island” OR “Navigator Islands” OR “Sao Tome” OR “Saudi Arabia” OR Senegal OR Serbia OR Montenegro OR Seychelles OR “Sierra Leone” OR Singapore OR “Sri Lanka” OR Ceylon OR “Solomon Islands” OR Somalia OR “South Africa” OR Sudan OR Suriname OR Surinam OR Swaziland OR Syria OR Tajikistan OR Tadzhikistan OR Tadjikistan OR Tadzhik OR Tanzania OR Thailand OR Togo OR “Togolese Republic” OR Tokelau OR Tonga OR Trinidad OR Tobago OR Tunisia OR Turkey OR Turkmenistan OR Turkmen OR “Turks Caicos” OR “Turks and Caicos” OR Tuvalu OR Uganda OR “United Arab Emirates” OR Uruguay OR Uzbekistan OR Uzbek OR Vanuatu OR “New Hebrides” OR Venezuela OR Vietnam OR “Viet Nam” OR “Virgin Islands” OR “West Bank” OR Yemen OR Yugoslavia OR Zambia OR Zimbabwe**) OR AB (**Afghanistan OR Algeria OR Angola OR Anguilla OR Antigua OR Barbuda OR Argentina OR Armenia OR Armenian OR Aruba OR Azerbaijan OR Bahamas OR Bahrain OR Bangladesh OR Barbados OR Benin OR Byelarus OR Byelorussian OR Belarus OR Belorussian OR Belorussia OR Belize OR Bhutan OR Bolivia OR Botswana OR Brazil OR Brunei OR “Burkina Faso” OR “Burkina Fasso” OR “Upper Volta” OR Burundi OR Urundi OR Cambodia OR “Khmer Republic” OR Kampuchea OR Cameroon OR Cameroons OR Cameron OR Camerons OR “Cape Verde” OR “Cayman Islands” OR “Central African Republic” OR Chad OR Chile OR China OR Colombia OR Comoros OR “Comoro Islands” OR Comores OR Mayotte OR Congo OR Zaire OR “Cook Islands” OR “Costa Rica” OR “Cote d'Ivoire” OR “Ivory Coast” OR Croatia OR Cuba OR Cyprus OR Djibouti OR “French Somaliland” OR Dominica OR “Dominican Republic” OR “East Timor” OR “East Timur” OR “Timor Leste” OR Ecuador OR Egypt OR “United Arab Republic” OR “El Salvador” OR Eritrea OR Ethiopia OR “Falkland Islands” OR “Las Malvinas” OR Fiji OR Gabon OR “Gabonese Republic” OR Gambia OR Gaza OR “Georgia Republic” OR “Georgian Republic” OR Ghana OR “Gold Coast” OR Greece OR Grenada OR Guatemala OR Guinea OR Guam OR Guadeloupe OR Guiana OR Guyana OR Haiti OR Honduras OR “Hong Kong” OR India OR Maldives OR Indonesia OR Iran OR Iraq OR Jamaica OR Jordan OR Kazakhstan OR Kazakh OR Kenya OR Kiribati OR Korea OR Kosovo OR Kuwait OR Kyrgyzstan OR Kirghizia OR “Kyrgyz Republic” OR Kirghiz OR Kirgizstan OR “Lao PDR” OR Laos OR Lebanon OR Lesotho OR Basutoland OR Liberia OR Libya OR Macau OR Madagascar OR “Malagasy Republic” OR Maldives OR Malaysia OR Malaya OR Malay OR Sabah OR Sarawak OR Malawi OR Nyasaland OR Mali OR Malta OR “Marshall Islands” OR Martinique OR Mauritania OR Mauritius OR “Agalega Islands” OR Mexico OR Micronesia OR “Middle East” OR Mongolia OR Montserrat OR Morocco OR Ifni OR Mozambique OR Myanmar OR Myanma OR Burma OR Namibia OR Nauru OR Nepal OR Niui OR “Netherlands Antilles” OR “New Caledonia” OR Nicaragua OR Niger OR Nigeria OR “Northern Mariana Islands” OR Oman OR Mayotte OR Muscat OR Pakistan OR Palau OR Palestine OR Panama OR Paraguay OR Peru OR Philippines OR Philipines OR Phillipines OR Phillippines OR Polynesia OR “Puerto Rico” OR Qatar OR Reunion OR Rwanda OR Ruanda OR “Saint Kitts” OR “St Kitts” OR Nevis OR “Saint Lucia” OR “St Lucia” OR “Saint Vincent” OR “St Vincent” OR Grenadines OR Samoa OR “Samoan Islands” OR “Navigator Island” OR “Navigator Islands” OR “Sao Tome” OR “Saudi Arabia” OR Senegal OR Serbia OR Montenegro OR Seychelles OR “Sierra Leone” OR Singapore OR “Sri Lanka” OR Ceylon OR “Solomon Islands” OR Somalia OR “South Africa” OR Sudan OR Suriname OR Surinam OR Swaziland OR Syria OR Tajikistan OR Tadzhikistan OR Tadjikistan OR Tadzhik OR Tanzania OR Thailand OR Togo OR “Togolese Republic” OR Tokelau OR Tonga OR Trinidad OR Tobago OR Tunisia OR Turkey OR Turkmenistan OR Turkmen OR “Turks Caicos” OR “Turks and Caicos” OR Tuvalu OR Uganda OR “United Arab Emirates” OR Uruguay OR Uzbekistan OR Uzbek OR Vanuatu OR “New Hebrides” OR Venezuela OR Vietnam OR “Viet Nam” OR “Virgin Islands” OR “West Bank” OR Yemen OR Yugoslavia OR Zambia OR Zimbabwe**) OR TI (**Afghanistan OR Algeria OR Angola OR Anguilla OR Antigua OR Barbuda OR Argentina OR Armenia OR Armenian OR Aruba OR Azerbaijan OR Bahamas OR Bahrain OR Bangladesh OR Barbados OR Benin OR Byelarus OR Byelorussian OR Belarus OR Belorussian OR Belorussia OR Belize OR Bhutan OR Bolivia OR Botswana OR Brazil OR Brunei OR “Burkina Faso” OR “Burkina Fasso” OR “Upper Volta” OR Burundi OR Urundi OR Cambodia OR “Khmer Republic” OR Kampuchea OR Cameroon OR Cameroons OR Cameron OR Camerons OR “Cape Verde” OR “Cayman Islands” OR “Central African Republic” OR Chad OR Chile OR China OR Colombia OR Comoros OR “Comoro Islands” OR Comores OR Mayotte OR Congo OR Zaire OR “Cook Islands” OR “Costa Rica” OR “Cote d'Ivoire” OR “Ivory Coast” OR Croatia OR Cuba OR Cyprus OR Djibouti OR “French Somaliland” OR Dominica OR “Dominican Republic” OR “East Timor” OR “East Timur” OR “Timor Leste” OR Ecuador OR Egypt OR “United Arab Republic” OR “El Salvador” OR Eritrea OR Ethiopia OR “Falkland Islands” OR “Las Malvinas” OR Fiji OR Gabon OR “Gabonese Republic” OR Gambia OR Gaza OR “Georgia Republic” OR “Georgian Republic” OR Ghana OR “Gold Coast” OR Greece OR Grenada OR Guatemala OR Guinea OR Guam OR Guadeloupe OR Guiana OR Guyana OR Haiti OR Honduras OR “Hong Kong” OR India OR Maldives OR Indonesia OR Iran OR Iraq OR Jamaica OR Jordan OR Kazakhstan OR Kazakh OR Kenya OR Kiribati OR Korea OR Kosovo OR Kuwait OR Kyrgyzstan OR Kirghizia OR “Kyrgyz Republic” OR Kirghiz OR Kirgizstan OR “Lao PDR” OR Laos OR Lebanon OR Lesotho OR Basutoland OR Liberia OR Libya OR Macau OR Madagascar OR “Malagasy Republic” OR Maldives OR Malaysia OR Malaya OR Malay OR Sabah OR Sarawak OR Malawi OR Nyasaland OR Mali OR Malta OR “Marshall Islands” OR Martinique OR Mauritania OR Mauritius OR “Agalega Islands” OR Mexico OR Micronesia OR “Middle East” OR Mongolia OR Montserrat OR Morocco OR Ifni OR Mozambique OR Myanmar OR Myanma OR Burma OR Namibia OR Nauru OR Nepal OR Niui OR “Netherlands Antilles” OR “New Caledonia” OR Nicaragua OR Niger OR Nigeria OR “Northern Mariana Islands” OR Oman OR Mayotte OR Muscat OR Pakistan OR Palau OR Palestine OR Panama OR Paraguay OR Peru OR Philippines OR Philipines OR Phillipines OR Phillippines OR Polynesia OR “Puerto Rico” OR Qatar OR Reunion OR Rwanda OR Ruanda OR “Saint Kitts” OR “St Kitts” OR Nevis OR “Saint Lucia” OR “St Lucia” OR “Saint Vincent” OR “St Vincent” OR Grenadines OR Samoa OR “Samoan Islands” OR “Navigator Island” OR “Navigator Islands” OR “Sao Tome” OR “Saudi Arabia” OR Senegal OR Serbia OR Montenegro OR Seychelles OR “Sierra Leone” OR Singapore OR “Sri Lanka” OR Ceylon OR “Solomon Islands” OR Somalia OR “South Africa” OR Sudan OR Suriname OR Surinam OR Swaziland OR Syria OR Tajikistan OR Tadzhikistan OR Tadjikistan OR Tadzhik OR Tanzania OR Thailand OR Togo OR “Togolese Republic” OR Tokelau OR Tonga OR Trinidad OR Tobago OR Tunisia OR Turkey OR Turkmenistan OR Turkmen OR “Turks Caicos” OR “Turks and Caicos” OR Tuvalu OR Uganda OR “United Arab Emirates” OR Uruguay OR Uzbekistan OR Uzbek OR Vanuatu OR “New Hebrides” OR Venezuela OR Vietnam OR “Viet Nam” OR “Virgin Islands” OR “West Bank” OR Yemen OR Yugoslavia OR Zambia OR Zimbabwe**) )** |

| Proquest theses and dissertations global |
| --- |
| $ for wildcards (can be any letter or nothing)  * for truncation  Both can be used with quotation marks |
| Search date: 24 September, 2019 |
| Fields searched: anywhere but full text |
| noft((“standard precautions” OR “infection control” OR “infection prevention” OR IPC OR asepsis OR aseptic OR sterile OR sterility OR sterilization OR sterilisation OR “personal protective equipment” OR PPE OR “splash protection” OR “respiratory protection” OR mask$ OR glove$ OR gown$ OR scrubs OR goggles OR “eye protection” OR “face shield$” OR respirator$ OR Sharps OR needle$ OR syringe$ OR Water OR Sanitation OR Sanitary OR Plumbing OR Sewage OR Sewer* OR Latrine$ OR Toilet$ OR Hygiene OR Hygienic OR Shower$ OR Soap$ OR detergent$ OR Handwashing OR “hand washing” OR “hand hygiene” OR Laundry OR bedsheet$ OR bedding OR linen$ OR Waste$ OR Landfill$ OR Dump$ OR Drainage OR Wastewater OR “waste water” OR disposal OR lighting OR light$ OR “vector control” OR Vectors OR pest$ OR rodent$ OR rat$ OR insect$ OR vermin OR infest* OR fly OR flies OR mosquito OR mosquitoes OR Surface$ OR fomite$ OR Chlorine OR Disinfect* OR Cleaners OR cleaning OR cleanliness OR janitor* OR housekeep* “nosocomial infection$” OR “healthcare associated infection$” OR “health care associated infection$” OR “healthcare acquired infection$” OR “health care acquired infection$” OR HCAI OR HAI OR sepsis OR septic)) AND noft((healthcare OR “health care” OR hospital$ OR clinic$ OR “health facilit*” OR “health center$” OR healthcenter$ OR healthcentre$ OR “health centre$” OR “health post$” OR healthpost$ OR “health setting*” OR “medical facilit*” OR “medical center$” OR “medical centre$” OR “medical post$” OR “medical setting*” OR “delivery facilit*” OR “delivery center$” OR “delivery centre$” OR “delivery clinic$” OR “birth facilit*” OR “birth center$” OR “birth centre$” OR “birth clinic$” OR “matern* facilit*” OR “matern* center$” OR “matern* centre$” OR “matern* clinic$” OR “dental facilit*” OR “dental center$” OR “dental centre$” OR “dental clinic$” OR dispensary OR dispensaries)) AND noft((Cost$ OR costing OR economic OR budget* OR Price$ OR pricing OR fee$ OR tariff$ OR loan$ OR subsidy OR subsidies OR finance$ OR financial OR financing OR fund$ OR funding OR pay OR payment$ OR investment$ OR investing OR capital OR money OR monies OR expense$ OR accounting)) AND noft((Afghanistan OR Algeria OR Angola OR Anguilla OR Antigua OR Barbuda OR Argentina OR Armenia OR Armenian OR Aruba OR Azerbaijan OR Bahamas OR Bahrain OR Bangladesh OR Barbados OR Benin OR Byelarus OR Byelorussian OR Belarus OR Belorussian OR Belorussia OR Belize OR Bhutan OR Bolivia OR Botswana OR Brazil OR Brunei OR “Burkina Faso” OR “Burkina Fasso” OR “Upper Volta” OR Burundi OR Urundi OR Cambodia OR “Khmer Republic” OR Kampuchea OR Cameroon OR Cameroons OR Cameron OR Camerons OR “Cape Verde” OR “Cayman Islands” OR “Central African Republic” OR Chad OR Chile OR China OR Colombia OR Comoros OR “Comoro Islands” OR Comores OR Mayotte OR Congo OR Zaire OR “Cook Islands” OR “Costa Rica” OR “Cote d'Ivoire” OR “Ivory Coast” OR Croatia OR Cuba OR Cyprus OR Djibouti OR “French Somaliland” OR Dominica OR “Dominican Republic” OR “East Timor” OR “East Timur” OR “Timor Leste” OR Ecuador OR Egypt OR “United Arab Republic” OR “El Salvador” OR Eritrea OR Ethiopia OR “Falkland Islands” OR “Las Malvinas” OR Fiji OR Gabon OR “Gabonese Republic” OR Gambia OR Gaza OR “Georgia Republic” OR “Georgian Republic” OR Ghana OR “Gold Coast” OR Greece OR Grenada OR Guatemala OR Guinea OR Guam OR Guadeloupe OR Guiana OR Guyana OR Haiti OR Honduras OR “Hong Kong” OR India OR Maldives OR Indonesia OR Iran OR Iraq OR Jamaica OR Jordan OR Kazakhstan OR Kazakh OR Kenya OR Kiribati OR Korea OR Kosovo OR Kuwait OR Kyrgyzstan OR Kirghizia OR “Kyrgyz Republic” OR Kirghiz OR Kirgizstan OR “Lao PDR” OR Laos OR Lebanon OR Lesotho OR Basutoland OR Liberia OR Libya OR Macau OR Madagascar OR “Malagasy Republic” OR Maldives OR Malaysia OR Malaya OR Malay OR Sabah OR Sarawak OR Malawi OR Nyasaland OR Mali OR Malta OR “Marshall Islands” OR Martinique OR Mauritania OR Mauritius OR “Agalega Islands” OR Mexico OR Micronesia OR “Middle East” OR Mongolia OR Montserrat OR Morocco OR Ifni OR Mozambique OR Myanmar OR Myanma OR Burma OR Namibia OR Nauru OR Nepal OR Niui OR “Netherlands Antilles” OR “New Caledonia” OR Nicaragua OR Niger OR Nigeria OR “Northern Mariana Islands” OR Oman OR Mayotte OR Muscat OR Pakistan OR Palau OR Palestine OR Panama OR Paraguay OR Peru OR Philippines OR Philipines OR Phillipines OR Phillippines OR Polynesia OR “Puerto Rico” OR Qatar OR Reunion OR Rwanda OR Ruanda OR “Saint Kitts” OR “St Kitts” OR Nevis OR “Saint Lucia” OR “St Lucia” OR “Saint Vincent” OR “St Vincent” OR Grenadines OR Samoa OR “Samoan Islands” OR “Navigator Island” OR “Navigator Islands” OR “Sao Tome” OR “Saudi Arabia” OR Senegal OR Serbia OR Montenegro OR Seychelles OR “Sierra Leone” OR Singapore OR “Sri Lanka” OR Ceylon OR “Solomon Islands” OR Somalia OR “South Africa” OR Sudan OR Suriname OR Surinam OR Swaziland OR Syria OR Tajikistan OR Tadzhikistan OR Tadjikistan OR Tadzhik OR Tanzania OR Thailand OR Togo OR “Togolese Republic” OR Tokelau OR Tonga OR Trinidad OR Tobago OR Tunisia OR Turkey OR Turkmenistan OR Turkmen OR “Turks Caicos” OR “Turks and Caicos” OR Tuvalu OR Uganda OR “United Arab Emirates” OR Uruguay OR Uzbekistan OR Uzbek OR Vanuatu OR “New Hebrides” OR Venezuela OR Vietnam OR “Viet Nam” OR “Virgin Islands” OR “West Bank” OR Yemen OR Yugoslavia OR Zambia OR Zimbabwe)) |
