# Supplementary material for: Safe Healthcare Facilities: A Systematic Review on the Costs of Establishing and Maintaining Environmental Health in Facilities in Low- and Middle-Income Countries
Source: Int J Environ Res Public Health. 2021 Jan 19;18(2):817. doi: 10.3390/ijerph18020817 (PMC7833392; doi:10.3390/ijerph18020817)
Supplement: Supplementary file 1 [file ijerph-18-00817-s001.zip › Supplements/Supplementary Information File 6 - Quality scoring tool - EHS in HCF systematic review.docx]

**Quality scoring tool**

Definitions

**Costing framework:** a tool that describes and categorizes expenditures required for service provision, and that may be used to identify expected costs; frameworks may be developed internally for study purposes or referenced from existing literature

**Indicators of service quantity**: an indicator of patient volume or other facility characteristics that may serve as a proxy for service demand

**Direct measures of service quantity**: a direct measure of a service input or output

Cost categories

| **Cost category** | **Definition** |
| --- | --- |
| Capital hardware | Infrastructure or equipment purchases that are necessary to establish services or implement changes to service delivery method, which are not consumed during normal operation of the service |
| Capital maintenance | Expenses required to repair, rehabilitate, or otherwise maintain functionality of capital hardware, including labor costs required for these purposes |
| Capital software | Planning, procurement, and initial training costs associated with establishing new services or implementing changes to service delivery method |
| Recurrent training | Training required to ensure proper ongoing service provision regardless of changes to service delivery |
| Consumables | Products and supplies that are consumed during normal operation of the service |
| Personnel | Labor costs associated with normal operation of the service, including staff benefits |
| Direct support | Expenses required to supervise and monitor service provision to ensure safety and sustainability that support but do not have direct service outputs, such as auditing or developing management plans |
| Financing | Loan interest and other fees associated with financing the service |
| Contracted services | Fees paid to external providers to perform all or part of normal service operation, including multiple other cost categories, where expenses cannot be accurately disaggregated into categories above; where fees fall solely within another cost category described above, expenses should be included therein |

Scoring criteria

|  |  | **High quality (1 point)** | **Moderate quality (0.5 points)** | **Low quality (0.0 points)** |
| --- | --- | --- | --- | --- |
| **Context reporting** | Costing objective | Goal, objective, and/or research questions related to costing the specific EHS are reported | Goal, objective, and/or research questions related to costing are reported but are not EHS specific | Overall research goal is stated but costing objectives are not reported or are unclear OR no stated goals, objectives, or research questions |
|  | Facility description | All of the following are directly reported: facility type (e.g. teaching hospital, health post) and geographic location | At least one of the following are directly reported: facility type (e.g. teaching hospital, health post) or geographic location | Neither facility type nor geographic location are reported |
|  | Service description | Resources necessary to provide the service are described for each of the following categories: infrastructure and equipment, personnel, consumable products, trainings | Resources necessary to provide the service are reported for at least two of the following categories: infrastructure and equipment, personnel, consumable products, trainings | Resources necessary to provide the service are reported for less than two of the following categories: infrastructure and equipment, personnel consumable products, trainings |
|  | Service quantity indicators | At least one direct measure of the service quantity is reported | At least one indicator of service quantity is reported | No direct measures or indicators of service quantity are reported |
|  | Service quality indicators | A cost-effectiveness or cost-benefit analysis are conducted OR at least one quantitative indicator of service quality (i.e. a SMART indicator with all components defined, either within the paper or by an external organization or government body) is directly measured and reported | Service quality is narratively described without using quantitative indicators | Service quality is not reported or claimed without substantiation |
| **Costing reporting** | Units reporting | All relevant cost units are reported, including time period for operation costs; currency; year of costs data reported; foreign exchange rates; and inflation adjustments, where relevant | Currency and units for time-bound costs (where relevant) are reported, but other relevant information, such as year of costs data, foreign exchange rate, or inflation adjustment, are not reported or unclear | Currency units are not explicitly stated or ambiguous (e.g. “dollars” or $ versus USD or CAD) |
|  | Line item reporting | All line items included in cost calculations are reported | Categories of expenses included in costs are reported but not specific line items | No description of line items included in costs, or only a subset of line items are listed as examples but not representative of all items included |
|  | Analysis reporting | Methodology for calculating all costs is reported in full, including methodologies for annualizing capital costs, discounting, apportioning, and calculating unit costs | Methodologies for calculating costs are broadly reported but specific details (e.g. rate use for discounting or criteria used for apportioning) are not reported or methodologies for only a subset of steps are reported | Methodologies for calculating costs are not reported or unclear |
| **Costing methodology** | Framework use | A costing framework was used *a priori* to structure data collection | A costing framework was used but not selected *a priori* to structure data collection or timing of framework use was not specified as a priori | No costing framework was used |
|  | Data sources  [data sources refers specifically for costing the EHS; a study may use multiple data sources for non-EHS cost components but these are not relevant to quality scoring] | Cost data were collected using multiple methods, including at least one method that does not rely on participant recall (e.g. records review, structured observation); findings were compared across methods and/or collaboratively assessed by facility staff for validity | Cost data collection method uses a single source for each calculation input and does not rely on participant recall | Data collection relies on recall only or data source not reported/unclear |
|  | Coverage duration | Costs data covering > 12 months of expenses | Costs data cover > 6 to ≤ 12 months of expenses | Costs data cover ≤ 6 months of expenses or no coverage dates are reported |
|  | Cost category coverage | Costs include expenses from at least 4 of the following categories: direct support, capital hardware, capital software, capital maintenance, consumable products, personnel, training, financing | Costs include expenses from at least 3 of the following categories: direct support, capital hardware, capital software, capital maintenance, consumable products, personnel, training, financing | Costs include expenses from fewer than 3 of the following categories: direct support, capital hardware, capital software, capital maintenance, consumable products, personnel, training, financing |
